# Comparative efficacy and safety of second-line therapies for patients with advanced hepatocellular carcinoma: a systematic review and network meta-analysis of randomized controlled trials

**DOI:** 10.3389/fphar.2025.1697949

**Published:** 2025-11-20

**Authors:** Fengjiao Kang, Yin Wang, Fengqun Cai, Liuyun Wu, HuLin Wang, Lizhu Han, Qinan Yin, Lan Bai, Yuan Bian

**Affiliations:** 1 Department of Pharmacy, Personalized Drug Research and Therapy Key Laboratory of Sichuan Province, Sichuan Provincial People's Hospital, School of Medicine, University of Electronic Science and Technology of China, Chengdu, China; 2 Department of Pharmacy, Xinjiang Medical University Affiliated Traditional Chinese Medicine Hospital, Urumqi, China

**Keywords:** second-line therapy, overall survival, adverse events, network meta-analysis, hepatocellular carcinoma

## Abstract

**Background:**

The optimal second-line treatment for unresectable hepatocellular carcinoma (HCC) remains uncertain, given variability in efficacy and safety among available therapies. The comparative effectiveness and safety of second-line treatments for advanced HCC were methodically assessed in this network meta-analysis.

**Methods:**

A thorough search was conducted up until 20 February 2025, across the PubMed, Medline, Embase, Cochrane Central, and Web of Science databases to find randomized controlled trials (RCTs) evaluating second-line monotherapies (such as Ramucirumab, Regorafenib, Pembrolizumab, Cabozantinib, and Apatinib) in adults with advanced HCC. The main results comprised overall survival and progression-free survival; the supplementary results included objective response rate, disease control rate, and the occurrence of adverse events. A Bayesian random-effects network meta-analysis was employed for data synthesis, with interventions rated according to the SUCRA.

**Results:**

Eighteen RCTs involving 6,910 patients were analyzed. Ramucirumab (SUCRA: 69.2%), Regorafenib (67.6%), and Pembrolizumab (66.5%) significantly improved OS compared to control (mean difference [MD]: 2.79 months, 2.80 months, and 2.75 months, respectively). Apatinib (SUCRA: 93.0%; MD: 3.08 months), Cabozantinib (84.8%; MD: 2.65 months), and Regorafenib (48.9%; MD: 1.60 months) provided the most significant PFS benefits. Pembrolizumab (OR: 5.71, 95% CI, 2.71–12.04), Cabozantinib (OR: 5.38, 95% CI, 1.81–16.00), and Apatinib (OR: 5.32, 95% CI, 1.69–16.74) achieved superior ORR, while Apatinib, Cabozantinib, and Regorafenib had the highest DCR (OR: 3.92, 3.67, and 3.31, respectively). Pembrolizumab and Ramucirumab exhibited relatively lower incidences of severe adverse events (grade ≥3 AEs).

**Conclusion:**

Pembrolizumab and Ramucirumab had the most favorable balance of efficacy and tolerability among second-line treatments for advanced HCC and are indicated as optimal therapy alternatives to enhance clinical outcomes.

**Systematic Review Registration:**

https://www.crd.york.ac.uk/prospero/, identifier CRD420251010308

## 1 Introduction

Hepatocellular carcinoma (HCC) is a primary tumor originating from hepatocytes and is among the most malignant malignancies impacting the liver. Individuals with HCC frequently possess preexisting persistent liver conditions, such as cirrhosis and viral hepatitis, which may exacerbate disease management complexities (Haber et al., 2021). The prevalence of HCC is swiftly escalating in various global locations, indicating changes in risk variables, including chronic hepatitis B and C infections, metabolic syndrome, and alcohol use, and the progressive aging of the population (Devarbhavi et al., 2023). This growing burden not only places considerable strain on healthcare systems but also leads to substantial indirect costs (Younossi et al., 2019), such as loss of productivity and social support, thereby underscoring the need for improved therapeutic strategies.

Although curative approaches such as liver transplantation, surgical resection, and local ablation can yield favorable outcomes for patients diagnosed at an early stage, most individuals present with advanced disease at diagnosis, when these options are no longer feasible (Reig et al., 2022). According to the most recent international guideline, immune-based combinations including atezolizumab plus bevacizumab and durvalumab plus tremelimumab have become the preferred first-line systemic therapies for unresectable or advanced hepatocellular carcinoma (Singal et al., 2023; European Association for the Study of the Liver, 2018). Despite these advances, many patients still experience disease progression or intolerance after first-line treatment, highlighting the urgent need for effective and well-tolerated second-line options (Kudo et al., 2018; Bruix et al., 2017). However, real-world evidence indicates that only about 30%–45% of patients with advanced hepatocellular carcinoma are able to receive second-line systemic therapy after first-line treatment failure, mainly due to rapid disease progression, hepatic decompensation, or poor performance status (Wu et al., 2025; Seung et al., 2024). This limited transition underscores the importance of optimizing therapeutic efficacy and tolerability in the second-line setting to ensure that eligible patients achieve meaningful survival benefits. Second-line interventions, including tyrosine kinase inhibitors (TKIs), immune checkpoint inhibitors (ICIs), and other targeted agents, have therefore gained increasing importance in improving overall survival and quality of life for patients with advanced HCC (Yeo et al., 2025; Zhou et al., 2023). These therapies are integral for patients who have exhausted first-line options, offering an extended window of disease control and the potential for longer-term survival benefits.

Over the last decade, numerous randomized controlled studies have looked into various second-line medicines for advanced HCC, including apatinib, pembrolizumab, ramucirumab, cabozantinib, regorafenib and, among others. Each of these agents exhibits distinct pharmacological mechanisms; TKIs act by inhibiting multiple signaling pathways crucial for tumor growth, angiogenesis, and metastasis, whereas ICIs target immune checkpoint molecules to bolster antitumor immunity (Kudo et al., 2018; Zhu et al., 2015; Zhu et al., 2019). In parallel with clinical advances, several novel molecular and nanotechnology-based strategies have been developed to enhance treatment selectivity and immune responsiveness in HCC. For example, Li et al. synthesized a triantennary N-acetylgalactosamine–camptothecin prodrug that specifically targets hepatocytes via the asialoglycoprotein receptor, markedly improving solubility, tumor uptake, and immune activation (Li et al., 2025). Similarly, Zhang et al. introduced polyion complex micelles co-delivering an HDAC8 inhibitor and PD-L1 siRNA, achieving potent immune reprogramming, enhanced CD8^+^ T-cell infiltration, and robust tumor regression in HCC models (Zhao et al., 2025). These emerging approaches highlight a shift toward integrated molecular targeting and immunomodulatory strategies that complement current systemic therapies and inform future second-line treatment design.

Importantly, across the included trials, the vast majority of participants had received sorafenib as first-line systemic therapy, which was the global standard during the study periods (2015–2020). A few more recent trials also enrolled patients previously treated with lenvatinib or other immune-based first-line regimens, consistent with evolving clinical guidelines (Kudo et al., 2018). Clinical evidence supports the efficacy of these second-line strategies in delaying disease progression and improving survival; however, the relative benefits of different agents remain unclear. Existing meta-analyses have primarily focused on pairwise comparisons or limited treatment options, and there is a scarcity of comprehensive evidence directly comparing multiple second-line therapies to identify the optimal treatment approach (Li et al., 2023; Lu et al., 2024). Furthermore, heterogeneity in trial design, population characteristics, and outcome measures complicates the interpretation of results and highlights the need for a robust analytical method to integrate these diverse data.

In this context, an NMA provides the benefit of assessing many therapies simultaneously by pooling data from a range of randomized trials, regardless of whether direct head-to-head contrasts are missing (Leuch et al., 2016). Using evidence that is both indirect and direct, NMA can generate a hierarchical ranking of second-line treatments, facilitating the identification of the most promising regimens for clinical practice. Therefore, the objective of this research is to perform an extensive NMA, synthesizing evidence from high-quality randomized controlled trials to assess the relative effectiveness and safety of frequently utilized second-line therapies in HCC. By clarifying which regimens offer the greatest survival benefits with acceptable toxicity profiles, our findings seek to guide clinicians in optimizing options for therapy for individuals who have severe HCC and to influence additional studies in this swiftly progressing domain.

## 2 Methods

This systematic examination and NMA were carried out in compliance with the PRISMA 2020 guidelines and the PRISMA extension for NMA of healthcare interventions (Hutton et al., 2015; Page et al., 2021). The study did not call for ethical clearance or permission because it was a meta-analysis. The protocol was pre-registered in the PROSPERO database, under registration ID: CRD 420251010308.

### 2.1 Sources of data and the method of search

A thorough search for literature was performed across many databases, including Medline, Embase, PubMed, the Cochrane Central Register of Controlled Trials, and Web of Science, covering the period from the founding of each repository until 20 February 2025. The search terms included “hepatocellular carcinoma” along with second-line therapies such as “apatinib” “regorafenib” “ramucirumab” “pembrolizumab” and “cabozantinib” among others. The comprehensive screening technique, encompassing specific phrases and combinations, is accessible in Supplementary Material 1. Additionally, the reference lists of eligible studies and relevant systematic reviews published within the last 5 years were screened to ensure completeness.

Evaluations of titles, abstracts, and full texts were independently conducted by two reviewers according to the predefined inclusion and exclusion criteria. Inter-reviewer agreement was quantified using Cohen’s kappa statistic (κ = 0.87), indicating excellent consistency prior to study inclusion. Discrepancies were resolved through discussion and, when necessary, adjudicated by a third senior reviewer to ensure methodological rigor.

### 2.2 Selection of studies

Studies were considered if they matched the following requirements: (1) adult individuals (over 18 years old) suffering unresectable HCC and Child-Pugh scores of A or B; (2) patients in the experimental cohort received second-line immunotherapy or targeted monotherapy; (3) the only distinction among the experimental and placebo cohorts must be the receipt of second-line immunotherapy or targeted therapy; (4) one or more of the outcome measures listed below was documented: OS, PFS, TTP, ORR, DCR, incidence of AEs of all grades and grades 3–4, and occurrence of therapy cessation attributable to AEs; (5) study design is RCT. Articles were omitted if: (1) they utilized combination therapies with additional medicines, rather than monotherapy; (2) the treatment regimen was not clearly described; (3) the study did not include means or standard deviations, and the writers failed to respond to our data requests. In all eligible RCTs, participants had previously received at least one line of systemic therapy, predominantly sorafenib, as the prior standard of care, while a minority of recent studies included patients pretreated with lenvatinib or other immune-based regimens.

### 2.3 Extraction of data

EndNote X9 was used to organize studies that qualify in order to prevent duplication. Two impartial reviewers retrieved data, including research details (writer, title, year of publication), patient characteristics (age, gender), treatment interventions, and outcome measures (as detailed in Table 1). Missing means and standard deviations were imputed following the Cochrane Handbook (Higgins and Green, 2008). If the relevant data was not accessible via the procedures described above, the associated authors were notified at least 4 times within 6 weeks to ask for more information.

**TABLE 1:** Characteristics of studies and subjects included in the review.

Study	Study design	Country/region	Subjects (intervention/control)	Sex (male/female) (intervention/control)	Mean age (intervention/control)	Child-pugh	Intervention detail		Outcomes
							Intervention group	Control group	
Shao et al. (2022)	Double-blind RCT	China	104 (70/34)	55/15 vs. 31/3	57 ± 14 vs. 55 ± 11	Child-Pugh A5/A6	Ramucirumab 8 mg/kg every 2 weeks	Placebo	OS, PFS, ORR, DCR, AE, ≥3 AE
Zhu et al. (2019)	Double-blind RCT	20 countries (including the United States, China, Japan, and France)	292 (197/95)	154/43 vs. 79/16	64 ± 11 vs. 64 ± 11	Child-Pugh A5/A6	Ramucirumab 8 mg/kg every 2 weeks	Placebo plus best supportive care	OS, PFS, ORR, DCR, AE, ≥3 AE
Finn et al. (2018)	Double-blind RCT	27 countries (including the United States, South Korea, and France)	413 (278/135)	226/52 vs. 112/23	67 ± 18.2 vs. 65 ± 16.5	Child-Pugh A5/A6	Pembrolizumab 200 mg every 3 weeks	Placebo plus best supportive care	OS, PFS, ORR, DCR, AE, ≥3 AE
Bruix et al. (2017)	Double-blind RCT	Multinational (including China, the United States, France, and 21 countries)	573 (379/194)	333/46 vs. 171/23	64 ± 12.6 vs. 62 ± 9.6	Child-Pugh A/B	Regorafenib 160 mg daily	Placebo	OS, PFS, ORR, DCR, AE, ≥3 AE
Chiang et al. (2021)	Double-blind RCT	United States of America	413 (276/137)	186/90 vs. 90/47	65	Child-Pugh A	Pembrolizumab 200 mg every 3 weeks	Placebo plus best supportive care	OS, PFS, ORR, DCR, AE, ≥3 AE
Finn et al. (2018)	Double-blind RCT	Multinational (including the United States, Europe, Asia, and 21 countries)	573 (379/194)	303/76 vs. 163/31	64 ± 16.3 vs. 64 ± 15.8	Child-Pugh A/B	Regorafenib 160 mg daily	Placebo	OS, PFS, ORR, DCR, AE, ≥3 AE
Kudo et al. (2017)	Double-blind RCT	Japan	93 (45/48)	35/10 vs. 43/5	66 ± 10 vs. 67 ± 14.2	Child-Pugh A/B	Ramucirumab 8 mg/kg every 2 weeks	Placebo plus best supportive care	OS, PFS, ORR, DCR, AE, ≥3 AE
Kudo et al. (2020a)	Double-blind RCT	Multinational (including the United States, Canada, Europe, and Asia)	539 (316/223)	264/52 vs. 183/40	62.3 ± 10.2 vs. 61.8 ± 10.4	Child-Pugh A	Ramucirumab 8 mg/kg every 2 weeks	Placebo	OS, PFS, ORR, DCR, AE, ≥3 AE
Kudo et al. (2020b)	Double-blind RCT	Japan	59 (41/18)	31/10 vs. 16/2	71 ± 11.2 vs. 68 ± 10.5	Child-Pugh A5/A6	Ramucirumab 8 mg/kg every 2 weeks	Placebo	OS, PFS, ORR, DCR, AE, ≥3 AE
Qin et al. (2021)	Double-blind RCT	China, Hong Kong, South Korea, Malaysia, Taiwan	453 (300/153)	257/43 vs. 126/27	54	Child-Pugh A	Pembrolizumab 200 mg every 3 weeks	Placebo	OS, PFS, ORR, DCR, AE, ≥3 AE
Reig et al. (2021)	Double-blind RCT	Multinational (including the United States, Europe, and Asia)	565 (283/282)	195/88 vs. 219/63	62 vs. 60	Child-Pugh A	Ramucirumab 8 mg/kg every 2 weeks	Placebo	OS, PFS, ORR, DCR, AE, ≥3 AE
Qin et al. (2021)	Double-blind RCT	China	400 (267/133)	223/38 vs. 116/16	51 ± 13.3 vs. 50 ± 13	Child-Pugh A/B	Apatinib 750 mg once daily	Placebo	OS, PFS, ORR, DCR, AE, ≥3 AE
Zhu et al. (2015)	Double-blind RCT	Multinational (including the United States, China, and Europe)	413 (277/136)	202/75 vs. 97/39	60 ± 15 vs. 61 ± 13	Child-Pugh A	Ramucirumab 8 mg/kg every 2 weeks	Placebo	OS, PFS, ORR, DCR, AE, ≥3 AE
Robin et al. (2020)	Double-blind RCT	Multinational (including the United States of America, Korea, France, Italy, Hong Kong, Australia, and others)	495 (331/164)	264/67 vs. 144/20	63.0 ± 16 vs. 63.5 ± 13	Child-Pugh A	Cabozantinib 60 mg daily	Placebo	OS, PFS, ORR, DCR, ≥3 AE
Anthony et al. (2022)	Double-blind RCT	Multinational (including America, Europe, Asia, Canada, *etc.*)	73 (51/22)	45/6 vs. 20/2	63.0 ± 12.8 vs. 64.5 ± 8.9	Child-Pugh A/B	Cabozantinib 60 mg daily	Placebo	OS, PFS, ORR, DCR, AE, ≥3 AE
Robin et al. (2021)	Double-blind RCT	Multinational (including Asia, Europe, Pacific and North America)	703 (468/235)	377/91 vs. 200/35	62.0 ± 17.9 vs. 65.1 ± 15.7	Child-Pugh A/B	Cabozantinib 60 mg daily	Placebo	OS, PFS, ORR, DCR, ≥3 AE
Abou-Alfa et al. (2018)	Double-blind RCT	Multinational (including Asia, Europe, Pacific and North America)	707 (470/237)	379/91 vs. 202/35	64 ± 16 vs. 64 ± 15.5	Child-Pugh A5/A6	Cabozantinib 60 mg daily	Placebo	OS, PFS, ORR, DCR, AE, ≥3 AE
Lu et al. (2017)	Single-blind RCT	China	42 (20/22)	16/4 vs. 17/5	56.1 ± 10.79 vs. 58.9 ± 9.38	Child-Pugh A/B	Apatinib 500 mg once daily	Transcatheter arterial chemoembolization	PFS, ORR, AE, ≥3 AE

OS: Overall survival, PFS: Progression-Free Survival, ORR: Objective response rate, DCR: Disease control rate, AE: Adverse event, ≥3AE: Grade 3 or Higher Adverse Event.

### 2.4 Bias risk assessment

Two reviewers used the updated Cochrane risk of bias tool to evaluate the risk of bias in the research included in the review (RoB 2) (Sterne et al., 2019). The randomization procedure, departures from the planned measures, lacking data on outcomes, outcome measurement, and the selection of the findings were the five areas that were the focus of the evaluation. Any disagreements were resolved by consensus or, if unresolved, referred to a third reviewer.

### 2.5 Data analysis

The data was analyzed utilizing Stata 17.0. An NMA was performed out to assess the effectiveness of several second-line therapies for advanced HCC. Network graphs were produced to visually represent the links between various treatments, verifying that the network design was applicable. Clinical heterogeneity was taken into consideration, and within- and between-study changes were explained using a random-effects model. Mean differences (MD) with 95% CIs were employed to standardize data for continuous outcomes like OS and PFS, while OR with 95% CIs were used for dichotomous results like ORR, AEs, and DCR. Stata was used to do Bayesian NMA, with the “network” and “mvmeta” packages. The SUCRA was used to rank the therapies; larger SUCRA readings denoted greater relative treatment effectiveness. To identify bias in the publication, funnel plots and Egger’s test were used. A p-value of <0.05 indicated the presence of prejudice (Chaimani et al., 2013). All the tests used were two-sided, with a p-value of <0.05 indicating statistical significance.

To investigate potential effect modification by control group type, we conducted a subgroup analysis. Studies were categorized into two subgroups based on the control intervention: (1) pure placebo control, and (2) control involving placebo plus best supportive care or Transcatheter Arterial Chemoembolization (TACE). Subgroup differences were assessed qualitatively by comparing effect estimates and SUCRA rankings (Supplementary Material 2).

## 3 Results

### 3.1 Characteristics of included studies

**FIGURE 1:**
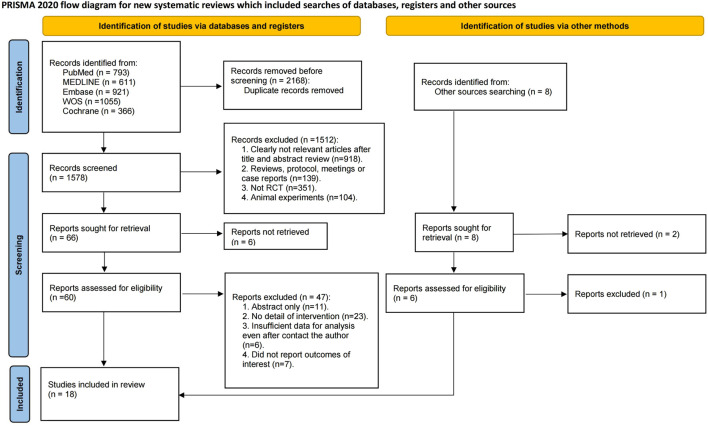
PRISMA Flow diagram of the search process for studies.

An aggregate of 3,746 documents were found via a preliminary electronic screening. Following the elimination of 2,168 duplicate data, 1,578 publications were evaluated based on their titles and abstracts. After excluding 1,512 research based on their titles and abstracts, 66 full-text papers were evaluated for inclusion. Ultimately, 18 RCTs were incorporated into the systematic review and NMA (Bruix et al., 2017; Zhu et al., 2015; Zhu et al., 2019; Abou-Alfa et al., 2018; Chiang et al., 2021; El-Khoueiry et al., 2022; Finn et al., 2018; Finn et al., 2020; Kelley et al., 2022; Kelley et al., 2020; Kudo et al., 2020a; Kudo et al., 2017; Kudo et al., 2020b; Lu et al., 2017; Qin et al., 2023; Qin et al., 2021; Reig et al., 2021; Shao et al., 2022), encompassing a total of 6,910 individuals with advanced HCC. It should be noted that the number of included studies and participants may vary slightly across specific outcomes due to incomplete reporting or different evaluable populations in certain trials. The flow of included studies is shown in Figure 1, and detailed characteristics are shown in Table 1.

The research articles included were produced from 2015 to 2022, with 2020 being the median publishing year. The total number of participants in the studies varied from 41 to 707 people, with a median of 413. The reported median age represents the median of study-level medians and is presented for descriptive purposes only. Participant ages ranged from 51 to 71 years, with 63 as the overall descriptive median. Seven trials investigated Ramucirumab, four Cabozantinib, three Pembrolizumab, two Apatinib, and two Regorafenib.

### 3.2 The network meta-analysis results

#### 3.2.1 Overall survival (OS)

**FIGURE 2:**
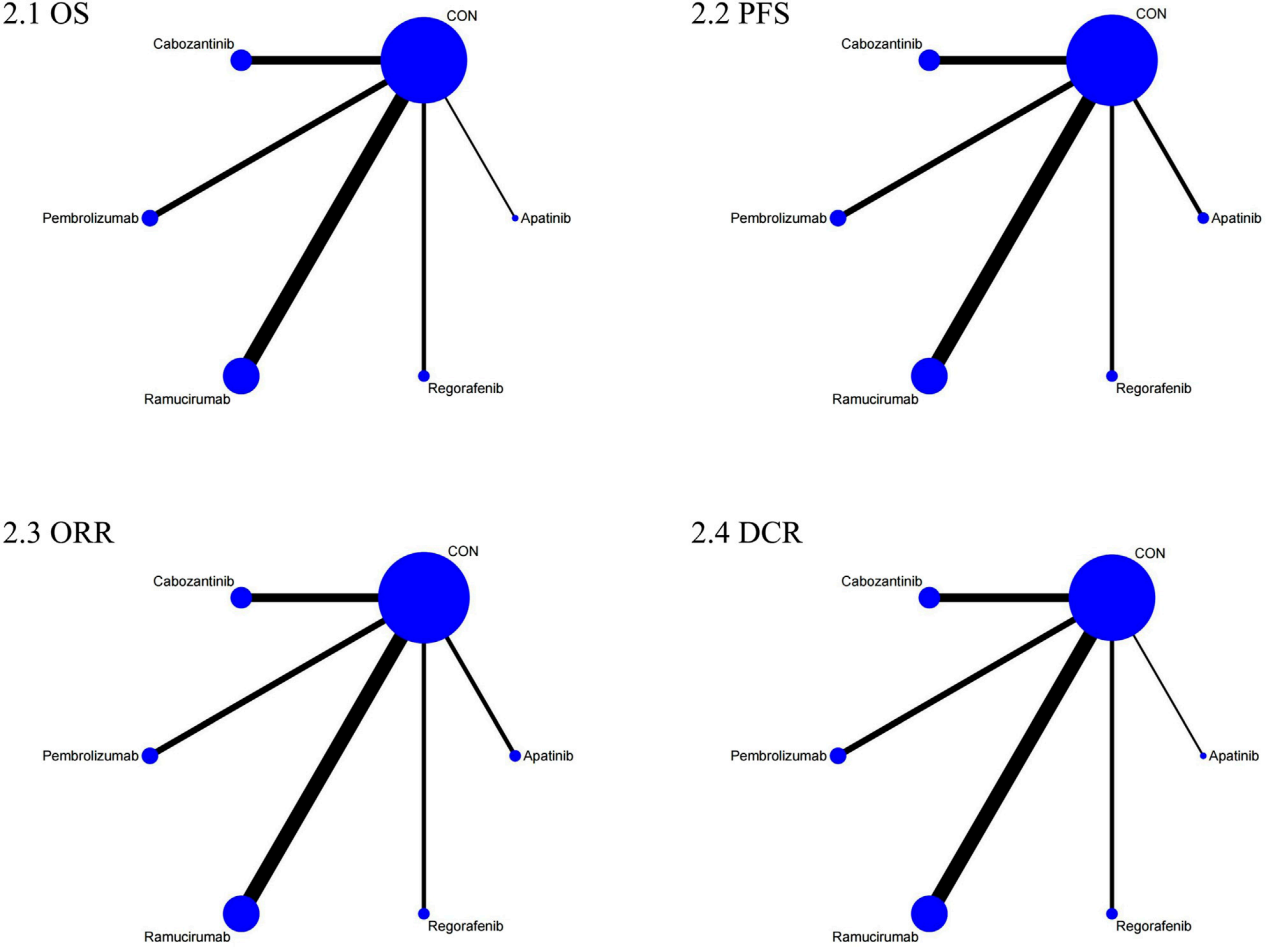
Network plot comparison of efficacy outcomes. 1: OS, 2: PFS, 3: ORR, 4: DCR.

**FIGURE 3:**
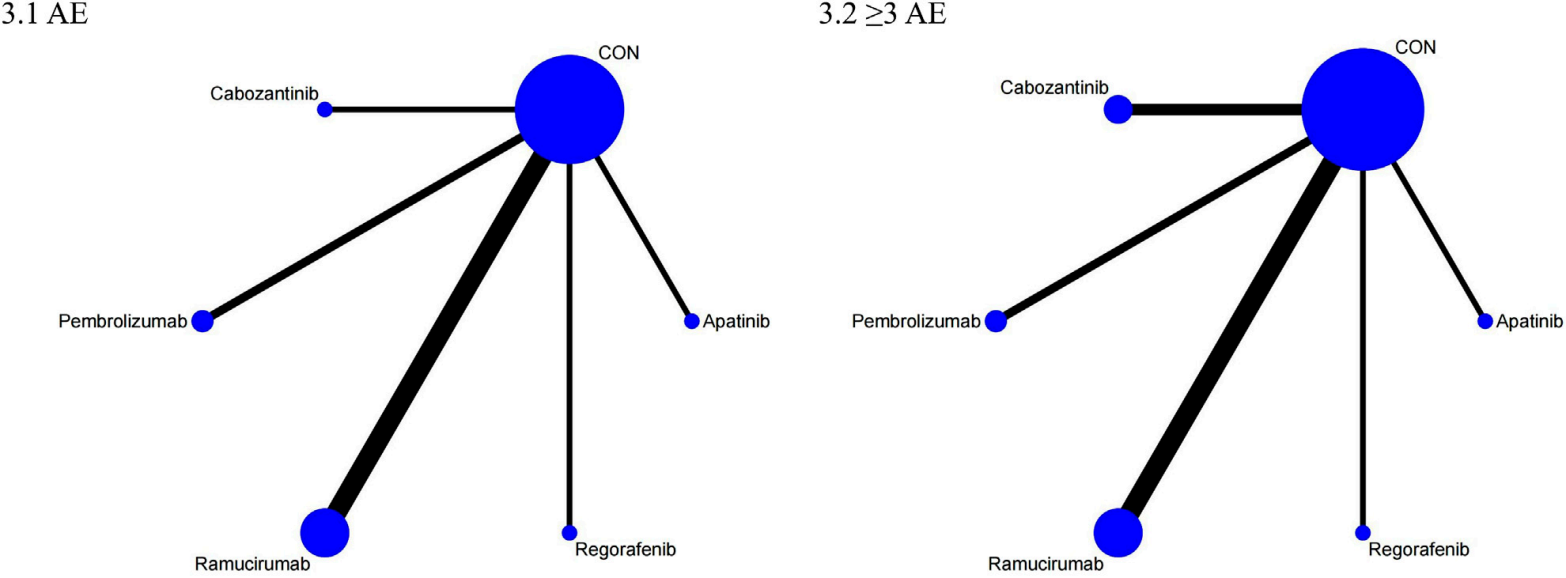
SUCRA probability ranking plot of efficacy outcomes. 1: OS, 2: PFS, 3: ORR, 4: DCR.

The NMA for OS comprised 17 investigations, encompassing an aggregate of 6,868 individuals who had advanced HCC, evaluating the effects of various second-line therapies on OS. Figure 2 illustrates the direct comparisons between treatments and the distribution of sample sizes. According to the SUCRA ranking (Figure 3), the top three treatments for improving OS were Ramucirumab (69.2%), Regorafenib (67.6%), and Pembrolizumab (66.5%). The worst-ranked treatment was the control group (2.0%).

As shown in Table 2, compared to the control cohort, Ramucirumab (MD = 2.79, 95% CI = 1.71, 3.87), Regorafenib (MD = 2.80, 95% CI = 0.79, 4.81), Pembrolizumab (MD = 2.75, 95% CI = 1.13, 4.38), and Cabozantinib (MD = 2.25, 95% CI = 0.80, 3.70) significantly improved OS.

**TABLE 2:** League table of efficacy.

OS					
Ramucirumab					
−0.01 (−2.29,2.27)	Regorafenib				
0.04 (−1.92,1.99)	0.05 (−2.53,2.63)	Pembrolizumab			
0.54 (−1.27,2.35)	0.55 (−1.93,3.02)	0.50 (−1.67,2.68)	Cabozantinib		
0.89 (−2.12,3.90)	0.90 (−2.55,4.35)	0.85 (−2.39,4.09)	0.35 (−2.80,3.51)	Apatinib	
**2.79 (1.71,3.87)**	**2.80 (0.79,4.81)**	**2.75 (1.13,4.38)**	**2.25 (0.80,3.70)**	1.90 (−0.90,4.70)	CON
PFS					
Apatinib					
0.42 (−1.09,1.94)	Cabozantinib				
1.48 (−0.17,3.12)	1.05 (−0.16,2.26)	Regorafenib			
**1.56 (0.14,2.99)**	**1.14 (0.25,2.02)**	0.08 (−1.02,1.19)	Ramucirumab		
**2.58 (1.03,4.12)**	**2.15 (1.09,3.22)**	1.10 (−0.16,2.36)	**1.01 (0.07,1.96)**	Pembrolizumab	
**3.08 (1.75,4.40)**	**2.65 (1.94,3.37)**	**1.60 (0.62,2.58)**	**1.52 (0.99,2.04)**	0.50 (−0.29,1.29)	CON
ORR					
Pembrolizumab					
1.06 (0.28,3.98)	Cabozantinib				
1.07 (0.27,4.22)	1.01 (0.21,4.92)	Apatinib			
1.27 (0.46,3.54)	1.20 (0.32,4.48)	1.19 (0.30,4.62)	Ramucirumab		
2.08 (0.71,6.14)	1.96 (0.51,7.51)	1.94 (0.48,7.78)	1.63 (0.56,4.78)	Regorafenib	
**5.71 (2.71,12.04)**	**5.38 (1.81,16.00)**	**5.32 (1.69,16.74)**	**4.48 (2.16,9.32)**	**2.74 (1.25,6.01)**	CON
DCR					
Apatinib					
1.07 (0.47,2.45)	Cabozantinib				
1.18 (0.49,2.86)	1.11 (0.60,2.05)	Regorafenib			
1.49 (0.67,3.32)	1.40 (0.86,2.28)	1.26 (0.71,2.25)	Ramucirumab		
2.10 (0.90,4.90)	**1.97 (1.12,3.45)**	1.78 (0.94,3.37)	1.41 (0.84,2.37)	Pembrolizumab	
**3.92 (1.87,8.20)**	**3.67 (2.52,5.36)**	**3.31 (2.04,5.39)**	**2.63 (1.92,3.60)**	**1.86 (1.23,2.82)**	CON

Bold values represent the statistically significant results with a p-value < 0.05.

We conducted subgroup analysis using two categories based on control interventions: (1) pure placebo control group, and (2) placebo combined with best supportive therapy or transarterial chemoembolization (TACE) control group. The statistical results showed essentially similar outcomes, further supporting our conclusions (Supplementary Material 3).

#### 3.2.2 Progression-free survival (PFS)

The NMA for PFS encompassed 18 trials including a total of 6,910 participants, assessing the effects of various second-line therapies on PFS. Figure 2 shows the direct comparisons between treatments and the distribution of sample sizes. According to the SUCRA ranking (Figure 3), the top three treatments for improving PFS were Apatinib (93.0%), Cabozantinib (84.8%), and Regorafenib (48.9%). The worst-ranked treatment was the control group (2.4%).

As shown in Table 2, in comparison to the control cohort, Apatinib (MD = 3.08, 95% CI = 1.75, 4.40), Cabozantinib (MD = 2.65, 95% CI = 1.94, 3.37), Regorafenib (MD = 1.60, 95% CI = 0.62, 2.58), and Ramucirumab (MD = 1.52, 95% CI = 0.99, 2.04) significantly improved PFS.

#### 3.2.3 Objective response rate (ORR)

The NMA for ORR encompassed 18 trials involving a total of 6,909 participants, assessing the effects of various second-line therapies on ORR. Figure 2 presents the direct comparisons between treatments and the distribution of sample sizes. According to the SUCRA ranking (Figure 3), the top three treatments for improving ORR were Pembrolizumab (73.1%), Cabozantinib (69.1%), and Apatinib (67.5%). The worst-ranked treatment was the control group (0.2%).

As shown in Table 2, all therapies markedly enhanced the ORR in comparison to the control cohort. Specifically, contrasted to the control cohort, Pembrolizumab (OR = 5.71, 95% CI = 2.71, 12.04), Cabozantinib (OR = 5.38, 95% CI = 1.81, 16.00), Apatinib (OR = 5.32, 95% CI = 1.69, 16.74), Ramucirumab (OR = 4.48, 95% CI = 2.16, 9.32), and Regorafenib (OR = 2.74, 95% CI = 1.25, 6.01) all significantly increased ORR.

#### 3.2.4 Disease control rate (DCR)

A total of 6,867 individuals from 17 trials assessing the impact of different second-line treatments on DCR were included in the NMA for DCR. Figure 2 presents the direct comparisons between treatments and the distribution of sample sizes. According to the SUCRA ranking (Figure 3), the top three treatments for improving DCR were Apatinib (80.0%), Cabozantinib (79.7%), and Regorafenib (69.1%). The worst-ranked treatment was the control group (0.0%).

As shown in Table 2, when contrasted to the control group, DCR was considerably increased by all therapies. Specifically, contrasted to the control cohort, Apatinib (OR = 3.92, 95% CI = 1.87, 8.20), Cabozantinib (OR = 3.67, 95% CI = 2.52, 5.36), Regorafenib (OR = 3.31, 95% CI = 2.04, 5.39), Ramucirumab (OR = 2.63, 95% CI = 1.92, 3.60), and Pembrolizumab (OR = 1.86, 95% CI = 1.23, 2.82) all significantly increased DCR.

#### 3.2.5 Adverse events (AEs)

**FIGURE 4:**
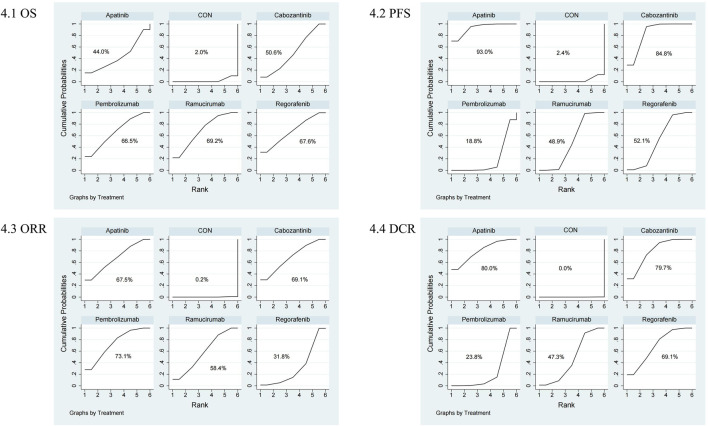
Network plot comparison of safety outcomes. 1: AE, 2: ≥3 AE.

**FIGURE 5:**
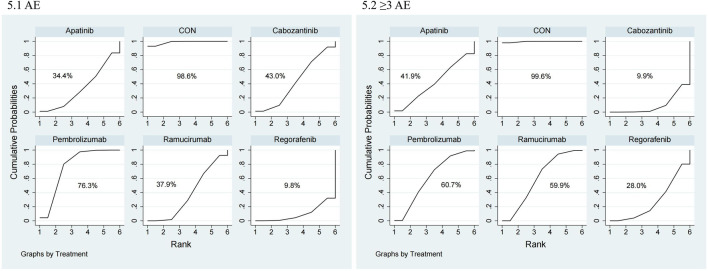
SUCRA probability ranking plot of safety outcomes. 1: AE, 2: ≥3 AE.

The NMA for AEs encompassed 16 trials including 5,703 individuals and evaluated the impacts of various second-line therapies on the occurrence of AEs. Figure 4 shows the direct comparisons between treatments and the distribution of sample sizes. According to the SUCRA ranking (Figure 5), the best 3 therapies for decreasing AEs comprised the control group (98.6%), Pembrolizumab (76.3%), and Cabozantinib (43.0%). The worst-ranked treatment was Regorafenib (9.8%).

As shown in Table 3, compared to Cabozantinib, Ramucirumab (OR = 0.25, 95% CI = 0.13, 0.46), Apatinib (OR = 0.21, 95% CI = 0.06, 0.80), and Regorafenib (OR = 0.11, 95% CI = 0.03, 0.39) substantially lowered the occurrence of AEs; Pembrolizumab significantly reduced AEs compared to Ramucirumab (OR = 0.41, 95% CI = 0.18, 0.97) and Regorafenib (OR = 0.18, 95% CI = 0.04, 0.75).

**TABLE 3:** League table of safety.

AE					
CON					
0.60 (0.33,1.09)	Pembrolizumab				
**0.28 (0.09,0.86)**	0.46 (0.13,1.65)	Cabozantinib			
**0.25 (0.13,0.46)**	**0.41 (0.18,0.97)**	0.90 (0.25,3.25)	Ramucirumab		
**0.21 (0.06,0.80)**	0.36 (0.08,1.55)	0.78 (0.13,4.56)	0.86 (0.17,4.30)	Apatinib	
**0.11 (0.03,0.39)**	**0.18 (0.04,0.75)**	0.39 (0.07,2.20)	0.43 (0.10,1.94)	0.50 (0.10,2.64)	Regorafenib

≥3 AE					
CON					
**0.54 (0.35,0.85)**	Pembrolizumab				
**0.53 (0.37,0.77)**	0.98 (0.56, 1.74)	Ramucirumab			
**0.44 (0.20,0.96)**	0.81 (0.33,1.99)	0.83 (0.35,1.95)	Apatinib		
**0.38 (0.24,0.61)**	0.70 (0.36,1.34)	0.71 (0.39,1.29)	0.86 (0.35,2.14)	Regorafenib	
**0.30 (0.21,0.44)**	0.56 (0.31,1.01)	**0.57 (0.33,0.97)**	0.69 (0.29,1.64)	0.80 (0.44,1.47)	Cabozantinib

Bold values represent the statistically significant results with a p-value < 0.05.

#### 3.2.6 ≥3 adverse events (≥3 AEs)

The NMA for ≥3 AEs encompassed 18 investigations, with an aggregate of 6,905 patients, assessing the impacts of various second-line therapies on the incidence of ≥3 AEs. Figure 4 presents the direct comparisons between treatments and the distribution of sample sizes. According to the SUCRA ranking (Figure 5), the top three treatments for reducing the incidence of ≥3 AEs were the control group (99.6%), Pembrolizumab (60.7%), and Ramucirumab (59.9%). The worst-ranked treatment was Cabozantinib (9.9%).

As shown in Table 3, the control group significantly reduced the incidence of ≥3 AEs in comparison to all second-line therapies, specifically contrasted to Pembrolizumab (OR = 0.54, 95% CI = 0.35, 0.85), Ramucirumab (OR = 0.53, 95% CI = 0.37, 0.77), Apatinib (OR = 0.44, 95% CI = 0.20, 0.96), Regorafenib (OR = 0.38, 95% CI = 0.24, 0.61), and Cabozantinib (OR = 0.30, 95% CI = 0.21, 0.44). Additionally, Ramucirumab substantially lowered the occurrence of ≥3 AEs in comparison to Cabozantinib (OR = 0.57, 95% CI = 0.33, 0.97).

### 3.3 Risk of bias

Among the 18 trials included in the analysis, 12 were evaluated to possess a low likelihood of bias, four were found to have certain issues, and 2 were determined to exhibit a high risk of bias. In terms of randomization, 16 studies had a low risk of bias, 1 study had significant issues, and one trial showed an elevated risk of bias. Regarding the discrepancies from the anticipated treatments, the entire 18 trials were evaluated as presenting a minimal risk of bias. Regarding missing outcome data, 16 trials had a minimal risk of bias, and 2 trials had elevated risk. For outcome measurement, 16 trials had a minimal risk of bias, and 2 investigations had certain issues. Lastly, for selective reporting, all 18 trials exhibited minimal risk of bias (Supplementary Material 4).

### 3.4 Inconsistency and publication bias

In order to evaluate the consistency of the NMA, the node-splitting method was employed to examine potential discrepancies within indirect and direct comparisons. The outcomes indicated no significant inconsistency across the network, as all p-values exceeded the predefined threshold for statistical significance (Supplementary Material 5). Additionally, the SIDE (separating indirect from direct evidence) test revealed no statistically significant differences across all outcomes, suggesting a coherent network structure. Furthermore, the consistent τ^2^ values across comparisons indicate that the level of heterogeneity was manageable and did not significantly impact the overall conclusions.

The evaluation of bias in publications was conducted through the utilization of funnel plots, which visually illustrate the distribution of effect estimates. The scatter points surrounding the vertical axis demonstrated varying degrees of symmetry, indicating the possibility of publication bias. Specifically, Supplementary Figure 6.6 exhibited relatively even point distribution, while Supplementary Figures 6.1–6.5 showed asymmetry, suggesting potential bias in some comparisons (Supplementary Material 6). However, the results of Egger’s test revealed no noteworthy small-study effects, with all p-values exceeding 0.05. This suggests that publication bias is unlikely to have a considerable influence on the overall conclusions drawn from this analysis.

## 4 Discussion

This NMA systematically evaluated the relative safety and effectiveness of various second-line therapies for individuals suffering from advanced HCC, providing critical evidence to inform clinical decision-making. The analysis revealed several key findings. Firstly, multiple second-line therapies demonstrated significant improvements in survival outcomes compared with placebo or standard care. Specifically, Ramucirumab, Regorafenib, and Pembrolizumab exhibited superior efficacy in prolonging OS, whereas Apatinib, Cabozantinib, and Regorafenib were particularly effective in improving PFS. Secondly, all second-line agents significantly improved both ORR and DCR compared to controls; notably, Pembrolizumab, Cabozantinib, and Apatinib were most effective for ORR improvement, and Apatinib, Cabozantinib, and Regorafenib demonstrated optimal performance regarding DCR. Thirdly, regarding safety, the control group consistently showed the lowest incidence of both overall AEs and ≥3 AEs. Among active treatments, Pembrolizumab and Ramucirumab displayed relatively favorable safety profiles, while Cabozantinib demonstrated a greater frequency of severe toxicity occurrences. Finally, when considering both therapeutic efficacy and safety comprehensively, Pembrolizumab and Ramucirumab emerged as optimal choices, demonstrating a balanced profile that combines substantial survival benefit with manageable toxicity. These findings suggest that Pembrolizumab and Ramucirumab may represent preferable second-line options for clinicians aiming to maximize patient survival while minimizing treatment-related risks in individuals suffering from advanced HCC. The included RCTs encompassed a diverse patient population with advanced HCC, reflecting the global epidemiology of the disease. Regarding liver function reserve, as assessed by the Child-Pugh score, the majority of studies predominantly enrolled patients with Child-Pugh A cirrhosis (specifically A5/A6 in many trials), indicating relatively preserved liver function. This is a common requirement for systemic therapy trials due to concerns about tolerability and altered drug metabolism in more compromised liver function. However, several trials also included patients with Child-Pugh B cirrhosis (e.g., Bruix et al., 2017; Finn et al., 2018; Kudo et al., 2017; Kudo et al. 2020a; Kudo et al. 2020c; Qin et al., 2021; Lu et al., 2017; Anthony et al., 2022; Robin et al., 2021). While the proportion of Child-Pugh B patients was typically smaller, their inclusion introduces heterogeneity related to liver function, potentially impacting drug metabolism, toxicity profiles, and overall prognosis. Our NMA attempted to account for this heterogeneity through random-effects modeling and subgroup analyses where feasible, but it remains a factor to consider when interpreting the overall results and applying them to clinical practice, especially for patients with poorer liver function. The underlying etiology of HCC in the included studies, while not uniformly reported in detail across all trials, aligns with the major global causes of the disease. Based on the geographical distribution of the trials (spanning Asia, Europe, North America, and multinational cohorts), it is reasonable to infer that the predominant etiologies included chronic hepatitis B virus (HBV) infection, particularly in Asian studies (e.g., Shao et al., 2022; Qin et al., 2021; Qin et al., 2023; Lu et al., 2017), and chronic hepatitis C virus (HCV) infection and alcohol-related liver disease, which are more common in Western populations (e.g., Finn et al., 2018; Bruix et al., 2017; Reig et al., 2021). Increasingly, non-alcoholic steatohepatitis (NASH)-related HCC was also likely represented, especially in trials recruiting from regions with high rates of metabolic syndrome. This etiological diversity is significant because the underlying liver disease can influence the tumor microenvironment, response to therapy (particularly immunotherapy), and the background risk of liver-related adverse events. For instance, HBV-related HCC might exhibit different immune profiles compared to HCV or NASH-related HCC, potentially affecting responses to immune checkpoint inhibitors like Pembrolizumab. While our analysis pooled results across these etiologies, consistent with the design of the included RCTs which typically did not select patients based on etiology, this represents another layer of heterogeneity inherent in the studied population. Future research exploring treatment efficacy stratified by etiology could provide further insights.

Consistent with clinical priorities in advanced HCC, OS and PFS represent critical endpoints in evaluating the efficacy of second-line therapeutic options, given their direct correlation with long-term survival and disease progression. In this NMA, Ramucirumab, Regorafenib, and Pembrolizumab has surfaced as the foremost effective agents for prolonging OS, whereas Apatinib, Cabozantinib, and Regorafenib demonstrated superior efficacy in extending PFS. The results align in part with earlier meta-analyses and RCTs, which have similarly underscored the clinical benefits of Ramucirumab and Regorafenib in terms of OS (Bruix et al., 2017; Wilke et al., 2014), though this study notably highlights the promising efficacy of Pembrolizumab, a result supported by the KEYNOTE-240 trial (Finn et al., 2018). Regarding PFS, Apatinib’s high ranking aligns with recent clinical trials demonstrating its significant antiangiogenic effect and notable efficacy in controlling tumor progression (Li et al., 2013; Ren et al., 2021).

It is also important to interpret these efficacy outcomes within the context of the epidemiologic heterogeneity among the included trials. Most participants across studies had preserved liver function, predominantly classified as Child-Pugh A or A5/A6, while a limited proportion of patients with Child-Pugh B status were included in select trials (Bruix et al., 2017; El-Khoueiry et al., 2022). Furthermore, the etiology of HCC varied geographically, with hepatitis B virus infection being the leading cause in Asian cohorts (Qin et al., 2021; Shao et al., 2022), and hepatitis C virus infection and alcohol-related liver disease being more prevalent in Western trials (Bruix et al., 2017; Abou-Alfa et al., 2018). Non-viral causes such as nonalcoholic steatohepatitis (NASH) also accounted for a smaller but notable subset of cases. These epidemiologic variations influence disease biology, drug metabolism, and therapeutic response, thereby contributing to the clinical and methodological heterogeneity observed across trials. Recognizing these differences is crucial for accurately interpreting the generalizability of our findings to broader patient populations. Another potential source of heterogeneity lies in the differences in control arms among the included studies. Most large-scale, global phase III trials—such as REACH, REACH-2, RESORCE, and KEYNOTE-240—employed placebo as the comparator, which has been the standard design for second-line monotherapy studies following sorafenib treatment failure. However, a few region-specific or earlier RCTs used active comparators such as best supportive care or transarterial chemoembolization (TACE) alone, reflecting local clinical practice patterns and resource availability (Zhu et al., 2019; Chiang et al., 2021; Finn et al., 2020; Kudo et al., 2017). These differences in comparator design could influence treatment effect estimates, as patients receiving supportive care or TACE might have different baseline liver function or performance status compared with those in placebo-controlled trials. Nonetheless, the consistency of efficacy and safety trends across trials indicates that the impact of such control-arm variability on the overall conclusions of this NMA is likely limited.

The superior OS benefits observed with Ramucirumab, Regorafenib, and Pembrolizumab are likely attributed to their distinct pharmacological mechanisms of operation. Ramucirumab specifically inhibits vascular endothelial growth factor receptor-2, directly addressing angiogenesis, an essential pathway for tumor development and dissemination in HCC (Zhu et al., 2019). Regorafenib demonstrates extensive antitumor activity by inhibiting angiogenesis of tumors, proliferation, and metastatic dissemination via various signaling pathways, including VEGFR, FGFR, and PDGFR (Bruix et al., 2017; Grothey et al., 2013). Pembrolizumab, which targets PD-1, improves T-cell-mediated tumor cytotoxicity, potentially facilitating sustained tumor regression and durable survival benefits (Reck et al., 2016; Gao et al., 2019). Conversely, Apatinib’s marked effectiveness in improving PFS may stem from its potent antiangiogenic activity via selective VEGFR-2 inhibition, effectively suppressing neovascularization crucial for HCC growth and metastasis, thereby delaying disease progression more evidently (Zhang et al., 2023). Taken together, these findings highlight distinct therapeutic mechanisms underlying the varied performance of these second-line agents, emphasizing the importance of aligning clinical strategies with specific therapeutic targets to optimize patient outcomes.

In addition to survival outcomes, ORR and DCR are important endpoints for evaluating tumor response and disease stabilization in advanced HCC, offering complementary perspectives on treatment efficacy. In this study, Pembrolizumab exhibited the most favorable therapeutic effects in terms of ORR, followed closely by Cabozantinib and Apatinib, whereas Apatinib demonstrated the highest efficacy in improving DCR, with Cabozantinib and Regorafenib ranking just behind. These findings partially align with previous randomized controlled trials, such as the KEYNOTE-240 trial, which highlighted Pembrolizumab’s potent effect in eliciting tumor response through immunological mechanisms involving enhanced antitumor immune activity mediated by PD-1 inhibition (Finn et al., 2020; Zhu et al., 2018). However, our study notably positions Apatinib prominently in terms of DCR, which contrasts with some earlier pairwise meta-analyses that identified Cabozantinib as having superior performance in controlling disease progression (Chen et al., 2024). The observed differences among trials may also be partly explained by variations in baseline liver function, etiology, and regional treatment practices. Patients with viral hepatitis or alcohol-related liver injury may respond differently to immune-based or antiangiogenic therapies due to underlying inflammatory and metabolic pathways. These inter-study differences underscore the importance of accounting for etiology- and liver-function–related factors when interpreting aggregated outcomes in HCC.

Differences in ORR and DCR rankings compared to OS and PFS outcomes may be attributed to distinct underlying biological mechanisms and pharmacological characteristics of the therapies investigated. ORR predominantly reflects direct tumor shrinkage and immediate response to treatment, influenced strongly by a therapy’s intrinsic antitumor potency, particularly the immunomodulatory capability of Pembrolizumab and the dual antiangiogenic and antitumor properties of Cabozantinib and Apatinib. Conversely, DCR, encompassing both tumor shrinkage and disease stabilization, captures a broader spectrum of therapeutic response, reflecting the capability of a treatment to halt or decelerate disease progression over a longer period. For instance, Apatinib’s marked efficacy in improving DCR likely stems from its selective and potent VEGFR-2 inhibition, significantly suppressing tumor angiogenesis and thereby stabilizing disease rather than achieving rapid tumor regression (Gao et al., 2019; Liao et al., 2019). On the other hand, Pembrolizumab, as an immune checkpoint inhibitor, directly activates cytotoxic immune responses, resulting in notable tumor shrinkage in responsive subsets of patients, thereby explaining its superior ORR performance (Adams et al., 2019). Such mechanistic distinctions underscore the necessity of adopting multidimensional outcome assessment when evaluating therapeutic efficacy, emphasizing the importance of tailored therapeutic decision-making according to specific patient profiles and treatment goals.

In addition to treatment efficacy, safety profiles, particularly the incidence of AEs and ≥3 AEs, represent critical determinants influencing therapeutic decisions in patients with advanced HCC. In this NMA, Pembrolizumab and Ramucirumab exhibited the most favorable safety profiles among second-line therapies, exhibiting a markedly reduced occurrence of overall AEs and grade ≥3 AEs in comparison to other agents investigated, notably Cabozantinib, which showed the highest risk of severe AEs. These findings align well with previous clinical trials, including KEYNOTE-240 and REACH-2, which highlighted that Pembrolizumab and Ramucirumab were associated with tolerable safety profiles characterized primarily by manageable immune-related and anti-angiogenic adverse effects, respectively (Zhu et al., 2019; Finn et al., 2020). Conversely, prior studies such as the CELESTIAL trial indicated that Cabozantinib, although efficacious, is often linked to increased occurrences of high blood pressure, hand-foot dermal reactions, tiredness, and gastrointestinal toxicities. Contributing to an elevated discontinuation rate due to adverse events (Abou-Alfa et al., 2018; Choueiri et al., 2015). The differences observed in safety profiles among these second-line therapies are intricately linked to their distinct pharmacodynamic mechanisms and off-target effects. Pembrolizumab exerts antitumor effects by selectively enhancing immune response, typically with fewer off-target toxicities than tyrosine kinase inhibitors (TKIs), which often affect multiple signaling pathways beyond the intended therapeutic target (O'Malley et al., 2022). Similarly, Ramucirumab, a monoclonal antibody targeting VEGFR-2 specifically, exhibits relatively precise targeting, resulting in fewer systemic adverse effects compared to multi-kinase inhibitors such as Cabozantinib or Regorafenib (Garon et al., 2014), which broadly inhibit multiple receptor kinases (VEGFR, PDGFR, FGFR, c-Kit), thus amplifying off-target toxicities (Wilke et al., 2014). Consequently, the pharmacological specificity of Pembrolizumab and Ramucirumab contributes substantially to their superior tolerability profiles, suggesting that these agents may offer significant clinical advantages in managing advanced HCC patients, particularly for those vulnerable to adverse events or with compromised liver function. The selection of second-line therapies should therefore balance therapeutic efficacy with patient tolerability, aligning clinical choices with individualized risk-benefit profiles to optimize patient-centered outcomes. Regarding study quality, most included trials showed low or moderate risk of bias. However, one study by Lu et al. had significant methodological concerns due to its single-center design, small sample size, lack of blinding, and limited allocation concealment, which may have introduced selection and performance bias (Lu et al., 2017). In addition, the RESORCE trial by Bruix et al., though generally robust, had a relatively high discontinuation rate related to adverse events and potential sponsor influence (Bruix et al., 2017). These factors were considered during quality assessment, and sensitivity analyses confirmed that they did not materially affect the overall findings.

Taking both efficacy and safety into comprehensive consideration, our analysis identified Pembrolizumab and Ramucirumab as the most favorable second-line therapeutic strategies for individuals diagnosed with advanced HCC. These two therapies demonstrated superior efficacy in prolonging OS, enhancing ORR, and effectively controlling illness progression, coupled with relatively favorable tolerability profiles characterized by lower incidences of severe adverse events compared to other investigated agents. Consequently, Pembrolizumab and Ramucirumab are recommended as preferred second-line therapeutic strategies for clinical practice in patients with unresectable HCC, especially when balancing treatment efficacy against the risks of toxicity. These results have significant ramifications for treating individuals with unresectable HCC, as clinicians can confidently prioritize these therapies to maximize patient survival benefits while simultaneously minimizing potential adverse effects. In the context of current global treatment patterns, these findings should be interpreted within the evolving therapeutic landscape. International guidelines, including those of the AASLD, EASL, and APASL, have recommended immune-based combinations such as atezolizumab plus bevacizumab or durvalumab plus tremelimumab as first-line regimens (Singal et al., 2023; Rose et al., 2024). Consequently, the choice of second-line therapy varies by region according to prior systemic exposure, drug accessibility, and healthcare resources. In Western countries, cabozantinib and regorafenib remain widely used, whereas pembrolizumab and ramucirumab are preferred for specific patient subgroups (Rajappa et al., 2022). In many Asian settings, apatinib is still frequently applied due to its availability and lower cost (Zheng et al., 2022). These regional differences suggest that while pembrolizumab and ramucirumab offer the most balanced efficacy and safety in this analysis, individualized treatment decisions should consider prior regimens and local clinical practice. Future studies are needed to clarify their optimal place in treatment sequencing worldwide. Furthermore, our results provide evidence-based guidance for informed clinical decision-making, aiding personalized treatment planning tailored to individual patient conditions, and ultimately contributing to improved patient-centered outcomes in advanced HCC.

This research presents several significant advantages. To begin with, the current NMA systematically integrates evidence from numerous high-quality RCTs to evaluate the safety and effectiveness profiles of different second-line treatments in advanced HCC. By simultaneously considering multiple treatment options through direct and indirect comparisons, our findings provide clinicians with robust, evidence-based guidance for selecting optimal second-line therapies. Second, our rigorous methodological approach—adhering strictly to PRISMA guidelines, employing comprehensive search strategies across multiple databases, and performing thorough bias assessments using the RoB 2 tool—ensures the validity and reliability of the synthesized evidence. However, several limitations should also be acknowledged. First, despite efforts to minimize heterogeneity through strict inclusion and exclusion criteria, inherent clinical and methodological heterogeneity—such as differences in patient demographics, liver function status, treatment regimens, and follow-up durations across trials—could have impacted the strength of our findings. In addition, descriptive demographic data such as age were summarized using study-level medians rather than patient-level data, and these should be interpreted as approximate indicators of overall population characteristics rather than pooled estimates. Secondly, our examination predominantly focused on monotherapy-based second-line treatments. This consequently restricts the applicability of the results to combination therapies, that are becoming more prevalent in modern clinical practice. Third, due to the intrinsic limitations of aggregated trial-level data, patient-level factors such as detailed tumor staging, biomarker profiles, and individual differences in treatment tolerability were not available for subgroup analysis, potentially obscuring variations in response among distinct patient populations. Future meta-analyses incorporating data pertaining to individual patients are essential to clarify these subtleties and substantiate our findings. Furthermore, we specifically addressed the concern regarding the composition of the control arm by performing a subgroup analysis. The distinction between pure placebo and more active control regimens (e.g., placebo plus best supportive care or TACE) is a recognized source of clinical heterogeneity. Our analysis revealed that the comparative effectiveness and safety rankings of the second-line therapies were generally robust across these control group subtypes. This consistency strengthens the credibility of our primary findings, as it indicates that the overall treatment effects observed in the main NMA are not driven predominantly by the choice of a particular control intervention. Nevertheless, we acknowledge this factor should be considered in the interpretation of network meta-analysis results.

## 5 Conclusion

This network meta-analysis of 18 randomized controlled trials including 6,910 patients with advanced hepatocellular carcinoma identified the most effective and tolerable second-line therapies. Ramucirumab, Regorafenib, and Pembrolizumab significantly improved overall survival, while Apatinib, Cabozantinib, and Regorafenib provided the greatest progression-free survival benefits.

Pembrolizumab and Ramucirumab showed the most favorable balance between efficacy and safety, offering substantial survival advantages with fewer severe adverse events. These agents represent the most promising second-line options for patients with advanced or unresectable HCC. Future studies should validate these findings and explore optimized combination strategies to enhance individualized treatment outcomes.

## Data Availability

The original contributions presented in the study are included in the article/Supplementary Material, further inquiries can be directed to the corresponding authors.
