# Supplementary material for: Comparative efficacy and safety of second-line therapies for patients with advanced hepatocellular carcinoma: a systematic review and network meta-analysis of randomized controlled trials
Source: Front Pharmacol. 2025 Nov 20;16:1697949. doi: 10.3389/fphar.2025.1697949 (PMC12675155; doi:10.3389/fphar.2025.1697949)
Supplement: Supplementary file 1 [file Supplementaryfile1.docx]

**Supplementary**

**Supplementary 1: Search Strategy**

***Search Strategy (PubMed):***

| #8 | Search: (((Carcinoma, Hepatocellular[MeSH Terms]) OR (((((("hepatocellular carcinoma"[Title/Abstract]) OR (hepatoma[Title/Abstract])) OR ("liver cell carcinoma"[Title/Abstract])) OR (HCC[Title/Abstract])) OR ("unresectable hepatocellular carcinoma"[Title/Abstract])) OR ("primary liver cancer"[Title/Abstract]))) AND (((Immunotherapy[MeSH Terms]) OR (Molecular Targeted Therapy[MeSH Terms])) OR (((((((((((((((immunotherapy[Title/Abstract]) OR ("immune checkpoint inhibitor"[Title/Abstract])) OR ("checkpoint inhibitor"[Title/Abstract])) OR ("molecular targeted therapy"[Title/Abstract])) OR ("targeted therapy"[Title/Abstract])) OR (apatinib[Title/Abstract])) OR (regorafenib[Title/Abstract])) OR (ramucirumab[Title/Abstract])) OR (pembrolizumab[Title/Abstract])) OR (cabozantinib[Title/Abstract])) OR ("second-line"[Title/Abstract])) OR ("second line"[Title/Abstract])))) AND ((randomized controlled trial[pt] OR controlled clinical trial[pt] OR randomized[tiab] OR placebo[tiab] OR clinical trials as topic[mesh:noexp] OR randomly[tiab] OR trial[ti]) NOT (animals[mh] NOT (humans[mh] AND animals[mh]))) |
| --- | --- |
| #7 | Search: (randomized controlled trial[pt] OR controlled clinical trial[pt] OR randomized[tiab] OR placebo[tiab] OR clinical trials as topic[mesh:noexp] OR randomly[tiab] OR trial[ti]) NOT (animals[mh] NOT (humans[mh] AND animals[mh])) |
| #6 | Search: ((Immunotherapy[MeSH Terms]) OR (Molecular Targeted Therapy[MeSH Terms])) OR (((((((((((((((immunotherapy[Title/Abstract]) OR ("immune checkpoint inhibitor"[Title/Abstract])) OR ("checkpoint inhibitor"[Title/Abstract])) OR ("molecular targeted therapy"[Title/Abstract])) OR ("targeted therapy"[Title/Abstract])) OR (apatinib[Title/Abstract])) OR (regorafenib[Title/Abstract])) OR (ramucirumab[Title/Abstract])) OR (pembrolizumab[Title/Abstract])) OR (cabozantinib[Title/Abstract])) OR ("second-line"[Title/Abstract])) OR ("second line"[Title/Abstract])) |
| #5 | Search: ((((((((((((((immunotherapy[Title/Abstract]) OR ("immune checkpoint inhibitor"[Title/Abstract])) OR ("checkpoint inhibitor"[Title/Abstract])) OR ("molecular targeted therapy"[Title/Abstract])) OR ("targeted therapy"[Title/Abstract])) OR (apatinib[Title/Abstract])) OR (regorafenib[Title/Abstract])) OR (ramucirumab[Title/Abstract])) OR (pembrolizumab[Title/Abstract])) OR (cabozantinib[Title/Abstract])) OR (nivolumab[Title/Abstract])) OR ("second-line"[Title/Abstract])) OR ("second line"[Title/Abstract]) |
| #4 | Search: (Immunotherapy[MeSH Terms]) OR (Molecular Targeted Therapy[MeSH Terms]) |
| #3 | Search: (Carcinoma, Hepatocellular[MeSH Terms]) OR (((((("hepatocellular carcinoma"[Title/Abstract]) OR (hepatoma[Title/Abstract])) OR ("liver cell carcinoma"[Title/Abstract])) OR (HCC[Title/Abstract])) OR ("unresectable hepatocellular carcinoma"[Title/Abstract])) OR ("primary liver cancer"[Title/Abstract])) |
| #2 | Search: ((((("hepatocellular carcinoma"[Title/Abstract]) OR (hepatoma[Title/Abstract])) OR ("liver cell carcinoma"[Title/Abstract])) OR (HCC[Title/Abstract])) OR ("unresectable hepatocellular carcinoma"[Title/Abstract])) OR ("primary liver cancer"[Title/Abstract]) |
| #1 | Search: Carcinoma, Hepatocellular[MeSH Terms] |

***Search Strategy (MEDLINE):***

1. *Carcinoma, Hepatocellular*.mp

2. exp *Liver Neoplasms*/

3. ("hepatocellular carcinoma" OR "hepatoma" OR "liver cell carcinoma" OR "HCC" OR "unresectable hepatocellular carcinoma" OR "primary liver cancer").mp

4. exp *Immunotherapy*/

5. exp *Molecular Targeted Therapy*/

6. ("immune checkpoint inhibitor" OR "checkpoint inhibitor" OR "molecular targeted therapy" OR "targeted therapy" OR "apatinib" OR "regorafenib" OR "ramucirumab" OR "pembrolizumab" OR "cabozantinib" OR "second-line" OR "second line").mp

7. exp *Randomized Controlled Trial/

8. exp *Clinical Trials as Topic*/

9. ("randomized controlled trial" OR "controlled clinical trial" OR "randomized" OR "placebo" OR "clinical trial" OR "randomly" OR "trial").mp

10. (crossover OR cross-over).mp

11. randomi$.mp

12. (random$ adj5 (assign$ OR allocat$ OR assort$ OR receiv$)).mp

13. 1 OR 2 OR 3

14. 4 OR 5 OR 6

15. 7 OR 8 OR 9 OR 10 OR 11 OR 12

16. 13 AND 14 AND 15

17. NOT ("animals"[MeSH] NOT ("humans"[MeSH] AND "animals"[MeSH]))

***Search Strategy (Embase):***

1. Carcinoma, Hepatocellular.mp
2. exp Liver Tumor/
3. ("hepatocellular carcinoma" OR "hepatoma" OR "liver cell carcinoma" OR "HCC" OR "unresectable hepatocellular carcinoma" OR "primary liver cancer").mp
4. exp Immunotherapy/
5. exp Molecular Targeted Therapy/
6. ("immune checkpoint inhibitor" OR "checkpoint inhibitor" OR "molecular targeted therapy" OR "targeted therapy" OR "apatinib" OR "regorafenib" OR "ramucirumab" OR "pembrolizumab" OR "cabozantinib" OR "second-line" OR "second line").mp
7. exp Randomized Controlled Trial/
8. exp Clinical Trial/
9. ("randomized controlled trial" OR "controlled clinical trial" OR "randomized" OR "placebo" OR "clinical trial" OR "randomly" OR "trial").mp
10. (crossover OR cross-over).mp
11. randomi$.mp
12. (random$ adj5 (assign$ OR allocat$ OR assort$ OR receiv$)).mp
13. 1 OR 2 OR 3
14. 4 OR 5 OR 6
15. 7 OR 8 OR 9 OR 10 OR 11 OR 12
16. 13 AND 14 AND 15
17. NOT ([animals]/lim NOT [humans]/lim)

***Search Strategy (Web of Science):***

| # 5 | #1 AND #2 AND #3 AND #4 Indexes=SCI-EXPANDED, SSCI, A&HCI, CPCI-S, CPCI-SSH, BKCI-S, BKCI-SSH, ESCI, CCR-EXPANDED, IC Timespan=All years |  |  |
| --- | --- | --- | --- |
| # 3 | TOPIC: ("randomized controlled trial*" OR "controlled clinical trial" OR "random*" OR "clinical trial*" OR "randomly" OR "trial" OR "cross-over studies" OR "clinic*") Indexes=SCI-EXPANDED, SSCI, A&HCI, CPCI-S, CPCI-SSH, BKCI-S, BKCI-SSH, ESCI, CCR-EXPANDED, IC Timespan=All years |  |  |
| # 3 | TOPIC: ("adverse events" OR "treatment-related toxicity" OR "safety profile" OR "grade 3-4 adverse events" OR "treatment discontinuation" OR "complication" OR "quality of life") Indexes=SCI-EXPANDED, SSCI, A&HCI, CPCI-S, CPCI-SSH, BKCI-S, BKCI-SSH, ESCI, CCR-EXPANDED, IC Timespan=All years |  |  |
| # 2 | TOPIC: ("Immunotherapy" OR "Molecular Targeted Therapy" OR "immune checkpoint inhibitor" OR "checkpoint inhibitor" OR "targeted therapy" OR "apatinib" OR "regorafenib" OR "ramucirumab" OR "pembrolizumab" OR "cabozantinib" OR "second-line" OR "second line") Indexes=SCI-EXPANDED, SSCI, A&HCI, CPCI-S, CPCI-SSH, BKCI-S, BKCI-SSH, ESCI, CCR-EXPANDED, IC Timespan=All years |  |  |
| # 1 | TOPIC: ("Carcinoma, Hepatocellular" OR "Hepatocellular carcinoma" OR "hepatoma" OR "liver cell carcinoma" OR "HCC" OR "unresectable hepatocellular carcinoma" OR "primary liver cancer") Indexes=SCI-EXPANDED, SSCI, A&HCI, CPCI-S, CPCI-SSH, BKCI-S, BKCI-SSH, ESCI, CCR-EXPANDED, IC Timespan=All years |  |  |

***Search Strategy (Cochrane):***

#1 MeSH descriptor: [Carcinoma, Hepatocellular] explode all trees

#2 MeSH descriptor: [Liver Neoplasms] explode all trees

#3 ("Hepatocellular carcinoma") OR ("hepatoma") OR ("liver cell carcinoma") OR ("HCC") OR ("unresectable hepatocellular carcinoma") OR ("primary liver cancer") in Trials (Word variations have been searched)

#4 MeSH descriptor: [Immunotherapy] explode all trees

#5 MeSH descriptor: [Molecular Targeted Therapy] explode all trees

#6 ("immune checkpoint inhibitor") OR ("checkpoint inhibitor") OR ("molecular targeted therapy") OR ("targeted therapy") OR ("apatinib") OR ("regorafenib") OR ("ramucirumab") OR ("pembrolizumab") OR ("cabozantinib") OR ("second-line") OR ("second line") in Trials (Word variations have been searched)

#7 MeSH descriptor: [Randomized Controlled Trial] explode all trees

#8 ("randomized controlled trial") OR ("controlled clinical trial") OR ("randomized") OR ("placebo") OR ("clinical trial") OR ("randomly") OR ("trial") in Trials (Word variations have been searched)

#9 MeSH descriptor: [Adverse Effects] explode all trees

#10 ("adverse events") OR ("treatment-related toxicity") OR ("safety profile") OR ("grade 3-4 adverse events") OR ("treatment discontinuation") OR ("complication") OR ("quality of life") in Trials (Word variations have been searched)

#11 #1 or #2 or #3

#12 #4 or #5 or #6

#13 #7 or #8

#14 #9 or #10

#15 #11 and #12 and #13 and #14

**
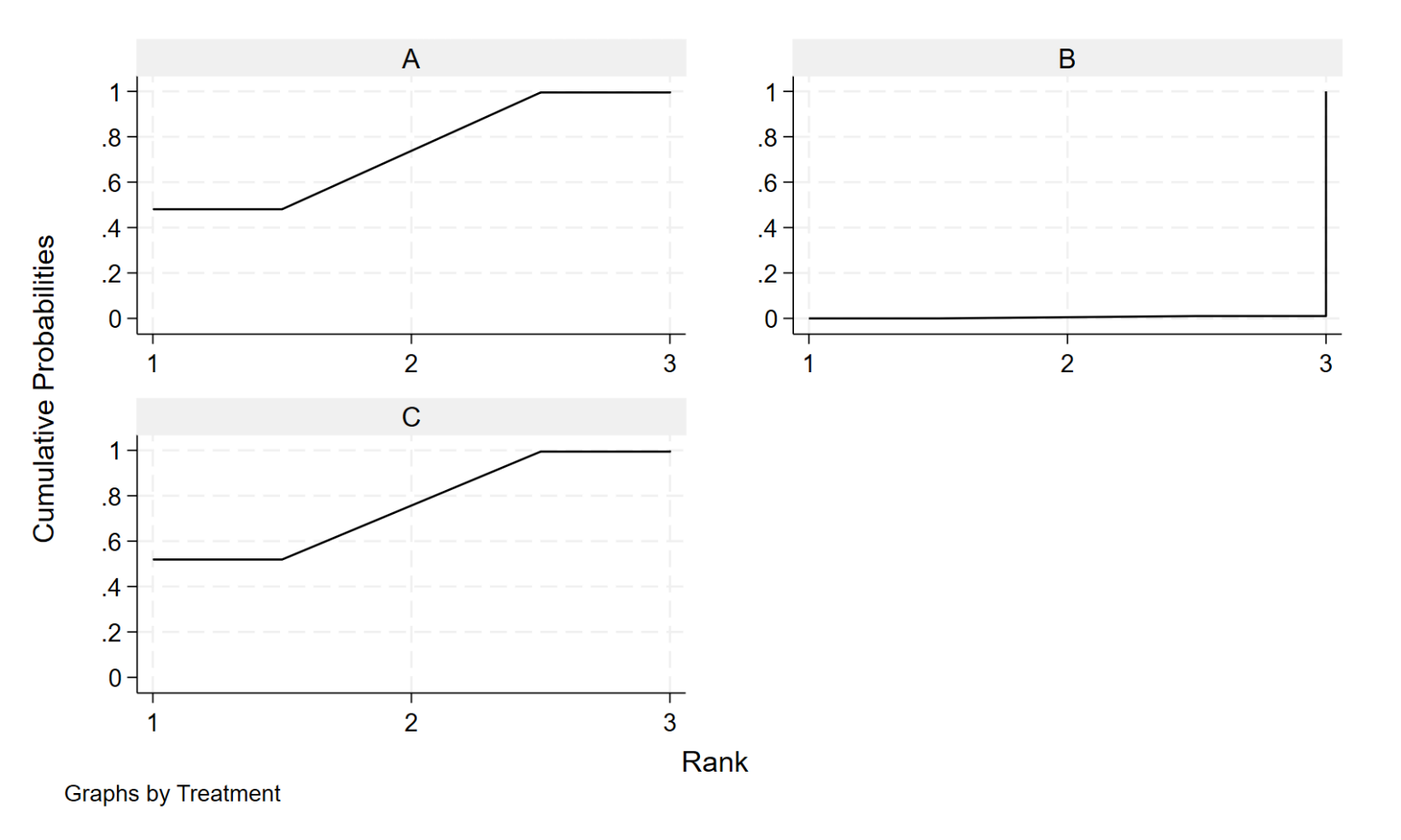
****Supplementary 2: SUCRA probability ranking plot of subgroup analysis**

Note: A: Ramucirumab; B: Placebo; C: Pembrolizumab

**Supplementary 3: Network plot comparison of subgroup analysis**

**
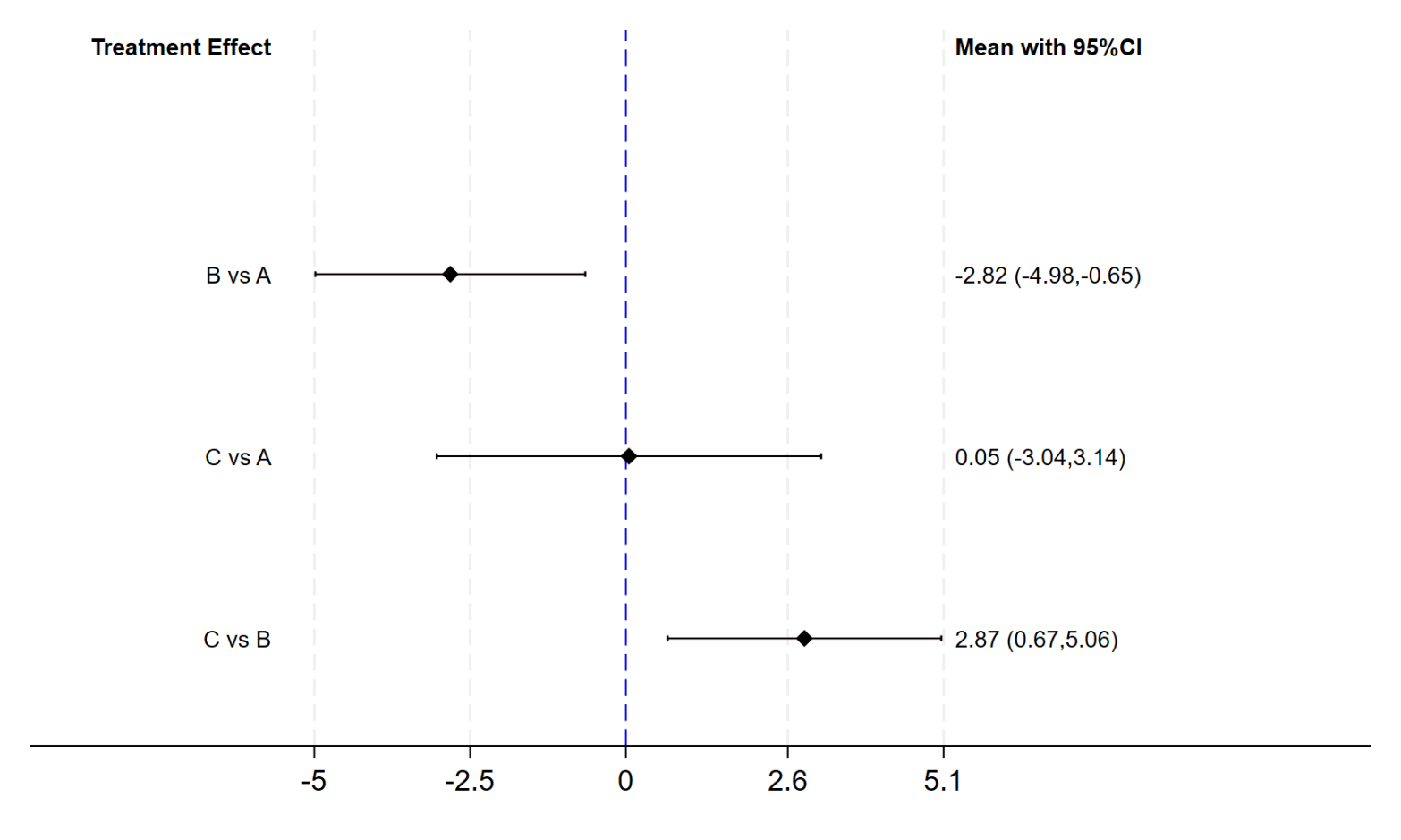
**

Note: A: Ramucirumab; B: Placebo; C: Pembrolizumab

# Supplementary 4: Risk of Bias

| **Author** | **Bias arising from the randomization process** | **Bias due to deviations from intended intervention** | **Bias due to missing outcome data** | **Bias in measurement of the outcome** | **Bias in selection of the reported result** | **Overall** |
| --- | --- | --- | --- | --- | --- | --- |
| Shao et al. (2022) | Low | Low | Low | Low | Low | Low |
| Zhu et al. (2019) | Low | Low | Low | Low | Low | Low |
| Finn et al. (2019) | Low | Low | Low | Low | Low | Low |
| Bruix et al. (2017) | High | Low | High | Low | Low | High |
| Chiang et al. (2021) | Low | Low | Low | Low | Low | Low |
| Finn et al. (2018) | Low | Low | Low | Low | Low | Low |
| Kudo et al. (2016) | Low | Low | Low | Some concerns | Low | Some concerns |
| Kudo et al. (2020a) | Low | Low | Low | Low | Low | Low |
| Kudo et al. (2020c) | Low | Low | Low | Low | Low | Low |
| Qin et al. (2022) | Low | Low | Low | Low | Low | Low |
| Reig et al. (2021) | Low | Low | Low | Some concerns | Low | Some concerns |
| Qin et al. (2021) | Low | Low | Low | Low | Low | Some concerns |
| Zhu et al. (2015) | Low | Low | Low | Low | Low | Low |
| Robin et al. (2020) | Low | Low | Low | Low | Low | Low |
| Anthony et al. (2022) | Low | Low | Low | Low | Low | Low |
| Robin et al. (2021) | Low | Low | Low | Low | Low | Low |
| Abou-Alfa et al. (2018) | Some concerns | Low | Low | Low | Low | Some concerns |
| Lu et al. (2017) | Low | Low | High | Low | Low | High |

# Supplementary 5: Evaluation of inconsistency

**Table 5.1 Details of SIDE splitting results (OS)**

| **Comparison** | **direct** | | **indir.** | | **Difference** | | **p-value** | **τ2** |
| --- | --- | --- | --- | --- | --- | --- | --- | --- |
|  | **Coef.** | **Std. Err.** | **Coef.** | **Std. Err.** | **Coef.** | **Std. Err.** |  |  |
| Apatinib vs CON | -1.9 | 1.431 | -1.701 | 131.374 | -0.199 | 131.382 | 0.999 | 1.415 |
| CON vs Cabozantinib | 2.252 | 0.739 | 3.034 | 740.275 | -0.782 | 740.275 | 0.999 | 1.415 |
| CON vs Pembrolizumab | 2.754 | 0.828 | 2.705 | 508.303 | 0.049 | 508.304 | 1 | 1.415 |
| CON vs Ramucirumab | 2.789 | 0.553 | 3.339 | 353.384 | -0.550 | 353.385 | 0.999 | 1.415 |
| CON vs Regorafenib | 2.8 | 1.024 | 2.877 | 1219.001 | -0.077 | 1219.002 | 1 | 1.415 |

**Table 5.2 Details of SIDE splitting results (PFS)**

| **Comparison** | **direct** | | **indir.** | | **Difference** | | **p-value** | **τ2** |
| --- | --- | --- | --- | --- | --- | --- | --- | --- |
|  | **Coef.** | **Std. Err.** | **Coef.** | **Std. Err.** | **Coef.** | **Std. Err.** |  |  |
| Apatinib vs CON | -3.077 | 0.676 | -0.731 | 45.708 | -2.346 | 45.713 | 0.959 | 0.696 |
| CON vs Cabozantinib | 2.652 | 0.364 | 5.242 | 423.144 | -2.59 | 423.144 | 0.995 | 0.696 |
| CON vs Pembrolizumab | 0.501 | 0.404 | 5.941 | 136.929 | -5.44 | 136.93 | 0.968 | 0.696 |
| CON vs Ramucirumab | 1.516 | 0.267 | 5.751 | 130.898 | -4.235 | 130.898 | 0.974 | 0.696 |
| CON vs Regorafenib | 1.6 | 0.498 | 5.638 | 667.793 | -4.038 | 667.793 | 0.995 | 0.696 |

**Table 5.3 Details of SIDE splitting results (ORR)**

| **Comparison** | **direct** | | **indir.** | | **Difference** | | **p-value** | **τ2** |
| --- | --- | --- | --- | --- | --- | --- | --- | --- |
|  | **Coef.** | **Std. Err.** | **Coef.** | **Std. Err.** | **Coef.** | **Std. Err.** |  |  |
| Apatinib vs CON | -1.671 | 0.585 | -1.154 | 325.991 | -0.517 | 325.991 | 0.999 | 0.402 |
| CON vs Cabozantinib | 1.683 | 0.556 | 3.465 | 1918.331 | -1.782 | 1918.331 | 0.999 | 0.402 |
| CON vs Pembrolizumab | 1.743 | 0.38 | 2.376 | 1046.128 | -0.634 | 1046.128 | 1 | 0.402 |
| CON vs Ramucirumab | 1.501 | 0.373 | 2.658 | 1013.229 | -1.158 | 1013.229 | 0.999 | 0.402 |
| CON vs Regorafenib | 1.009 | 0.4 | 2.843 | 1567.894 | -1.834 | 1567.894 | 0.999 | 0.402 |

**Table 5.4 Details of SIDE splitting results (DCR)**

| **Comparison** | **direct** | | **indir.** | | **Difference** | | **p-value** | **τ2** |
| --- | --- | --- | --- | --- | --- | --- | --- | --- |
|  | **Coef.** | **Std. Err.** | **Coef.** | **Std. Err.** | **Coef.** | **Std. Err.** |  |  |
| Apatinib vs CON | -1.366 | 0.377 | -0.664 | 159.906 | -0.701 | 159.907 | 0.997 | 0.299 |
| CON vs Cabozantinib | 1.301 | 0.193 | 2.25 | 663.959 | -0.949 | 663.959 | 0.999 | 0.299 |
| CON vs Pembrolizumab | 0.623 | 0.211 | 2.516 | 734.87 | -1.893 | 734.87 | 0.998 | 0.299 |
| CON vs Ramucirumab | 0.967 | 0.16 | 2.517 | 478.941 | -1.55 | 478.941 | 0.997 | 0.299 |
| CON vs Regorafenib | 1.198 | 0.248 | 2.368 | 906.808 | -1.17 | 906.808 | 0.999 | 0.299 |

**Table 5.5 Details of SIDE splitting results (AE)**

| **Comparison** | **direct** | | **indir.** | | **Difference** | | **p-value** | **τ2** |
| --- | --- | --- | --- | --- | --- | --- | --- | --- |
|  | **Coef.** | **Std. Err.** | **Coef.** | **Std. Err.** | **Coef.** | **Std. Err.** |  |  |
| Apatinib vs CON | -1.539 | 0.67 | -0.604 | 295.33 | -0.936 | 295.327 | 0.997 | 0.447 |
| CON vs Cabozantinib | 1.291 | 0.579 | 3.018 | 3237.222 | -1.727 | 3237.222 | 1 | 0.447 |
| CON vs Pembrolizumab | 0.511 | 0.303 | 2.917 | 992.29 | -2.406 | 992.29 | 0.998 | 0.447 |
| CON vs Ramucirumab | 1.393 | 0.316 | 2.97 | 769.266 | -1.577 | 769.266 | 0.998 | 0.447 |
| CON vs Regorafenib | 2.227 | 0.655 | 3.397 | 2340.022 | -1.171 | 2340.022 | 1 | 0.447 |

**Table 5.6 Details of SIDE splitting results (≥3AE)**

| **Comparison** | **direct** | | **indir.** | | **Difference** | | **p-value** | **τ2** |
| --- | --- | --- | --- | --- | --- | --- | --- | --- |
|  | **Coef.** | **Std. Err.** | **Coef.** | **Std. Err.** | **Coef.** | **Std. Err.** |  |  |
| Apatinib vs CON | -0.82 | 0.397 | -0.543 | 170.125 | -0.277 | 170.125 | 0.999 | 0.288 |
| CON vs Cabozantinib | 1.19 | 0.188 | 1.223 | 672.309 | -0.033 | 672.309 | 1 | 0.288 |
| CON vs Pembrolizumab | 0.61 | 0.231 | 1.412 | 917.643 | -0.802 | 917.643 | 0.999 | 0.288 |
| CON vs Ramucirumab | 0.628 | 0.187 | 1.504 | 486.83 | -0.875 | 486.83 | 0.999 | 0.288 |
| CON vs Regorafenib | 0.972 | 0.242 | 1.287 | 916.916 | -0.315 | 916.916 | 1 | 0.288 |

NA not available, direct Estimated treatment effect derived from direct evidence, indir. Estimated treatment effect derived from indirect evidence, Diff direct versus indirect, p-value of test for disagreement (direct versus indirect).

# Supplementary 6: Publication bias


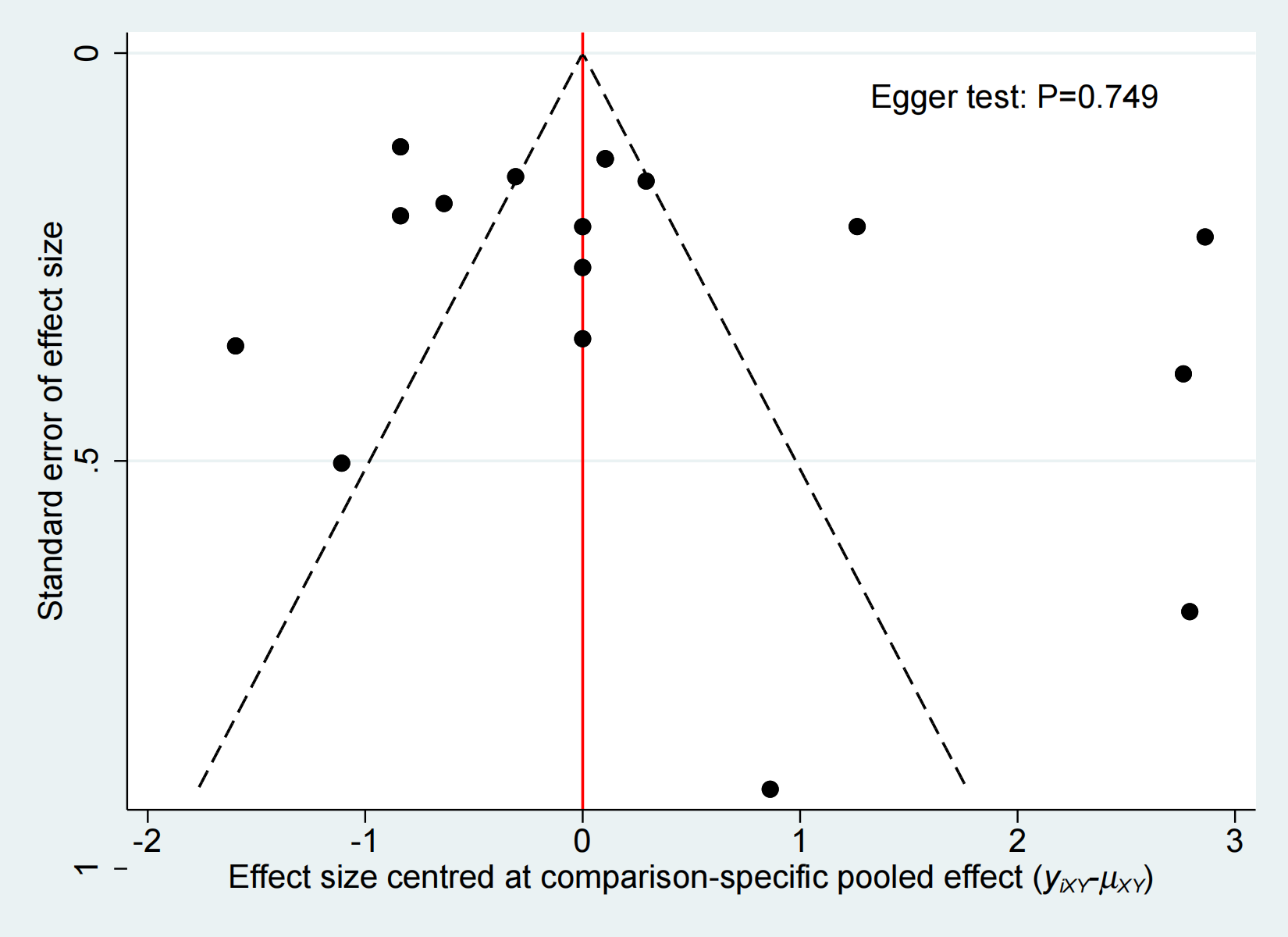


Figure 6.1 The funnel plot of OS. The result of Egger test showed the p=0.749.


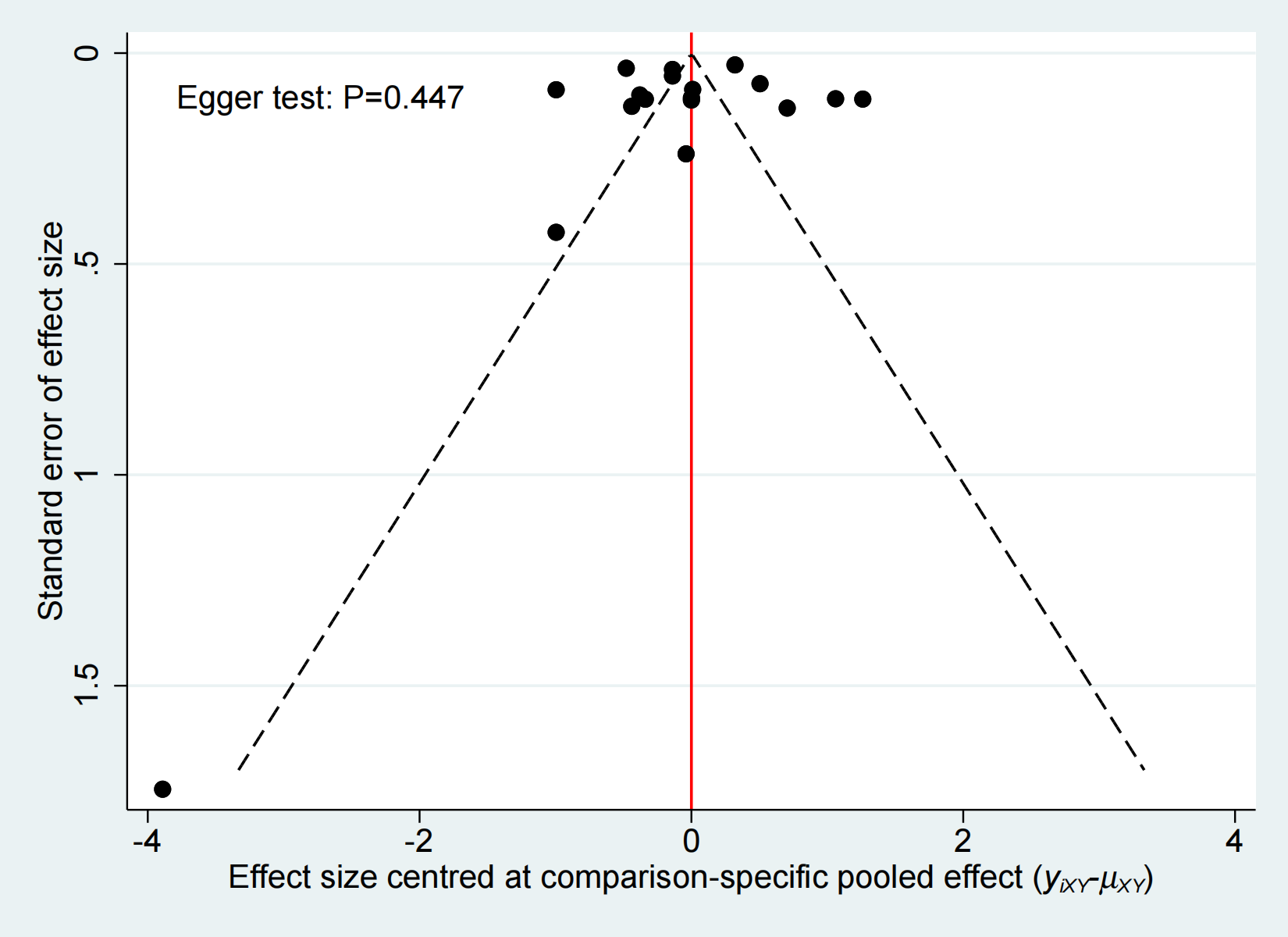


Figure 6.2 The funnel plot of PFS. The result of Egger test showed the p=0.447.


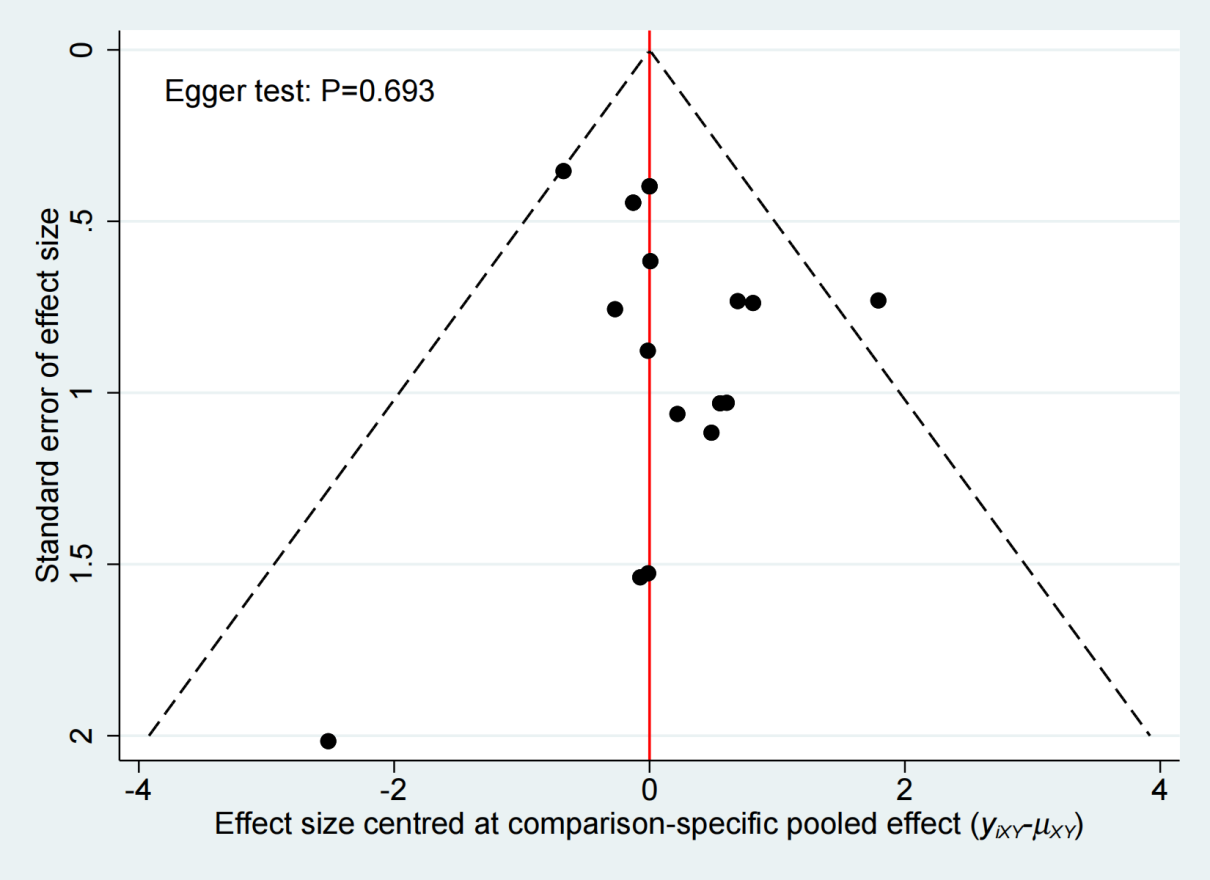


Figure 6.3 The funnel plot of ORR. The result of Egger test showed the p=0.693.


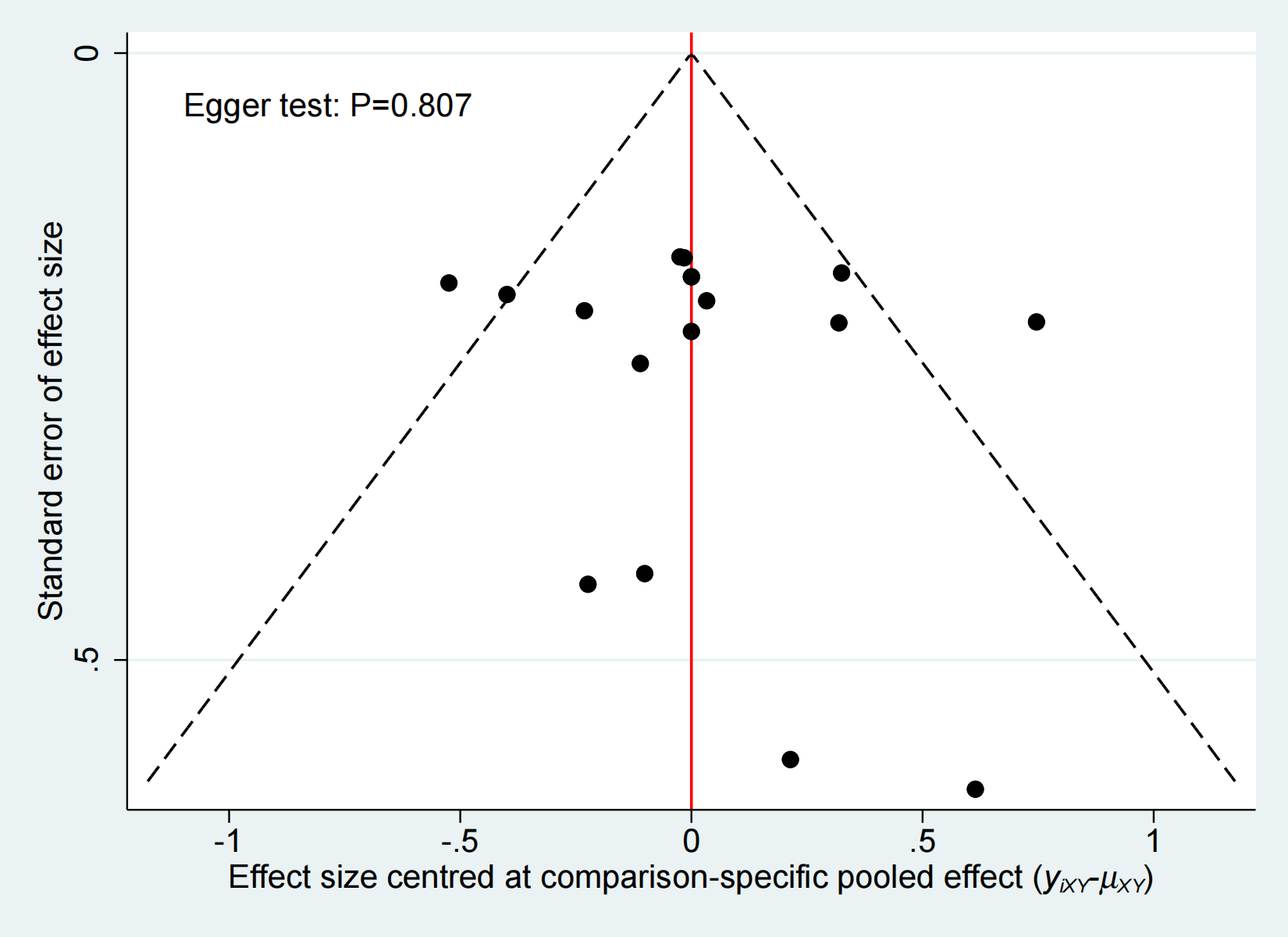


Figure 6.4 The funnel plot of DCR. The result of Egger test showed the p=0.807.


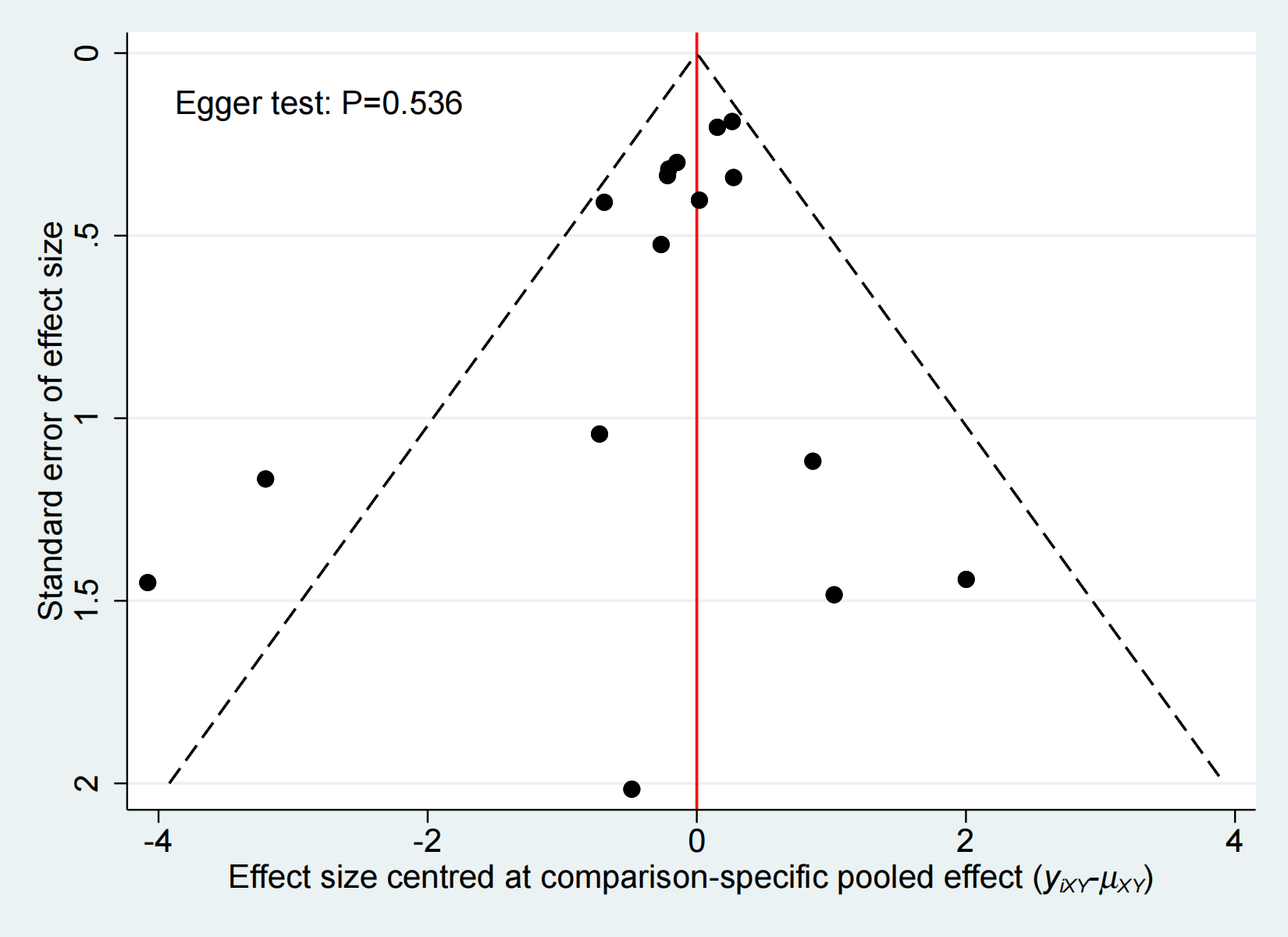


Figure 6.5 The funnel plot of AE. The result of Egger test showed the p=0.536.


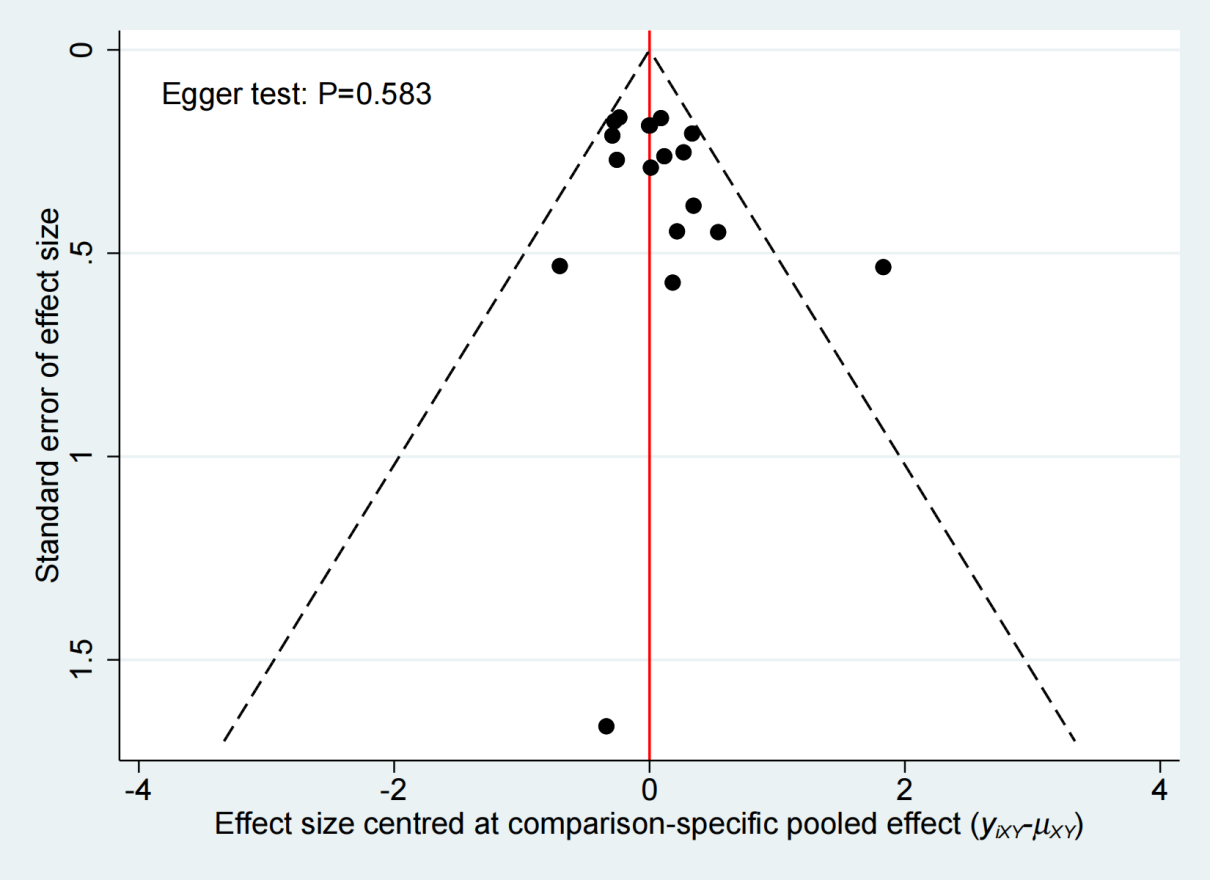


Figure 6.6 The funnel plot of ≥3AE. The result of Egger test showed the p=0.583.
